# Peptidic defective interfering gene nanoparticles against Omicron, Delta SARS-CoV-2 variants and influenza A virus in vivo

**DOI:** 10.1038/s41392-022-01138-0

**Published:** 2022-08-03

**Authors:** Hanjun Zhao, Chuyuan Zhang, Hoiyan Lam, Xinjie Meng, Zheng Peng, Man Lung Yeung, Jasper Fuk-Woo Chan, Kelvin Kai-Wang To, Kwok-Yung Yuen

**Affiliations:** 1 State Key Laboratory of Emerging Infectious Diseases, Li Ka Shing Faculty of Medicine, The University of Hong Kong, Hong Kong Special Administrative Region, China; 2 Department of Microbiology, School of Clinical Medicine, Li Ka Shing Faculty of Medicine, The University of Hong Kong, Hong Kong Special Administrative Region, China; 3 Centre for Virology, Vaccinology and Therapeutics, Hong Kong Science and Technology Park, Hong Kong Special Administrative Region, China; 4 Department of Infectious Disease and Microbiology, The University of Hong Kong-Shenzhen Hospital, Shenzhen, Guangdong Province China; 5 Carol Yu Centre for Infection, The University of Hong Kong, Pokfulam, Hong Kong Special Administrative Region, China; 6 Guangzhou Laboratory, Guangzhou, Guangdong Province China

**Keywords:** Drug delivery, Microbiology

## Abstract

Defective interfering genes (DIGs) are short viral genomes and interfere with wild-type viral replication. Here, we demonstrate that the new designed SARS-CoV-2 DIG (CD3600) can significantly inhibit the replication of SARS-CoV-2 including Alpha, Delta, Kappa and Omicron variants in human HK-2 cells and influenza DIG (PAD4) can significantly inhibit influenza virus replication in human A549 cells. One dose of influenza DIGs prophylactically protects 90% mice from lethal challenge of A(H1N1)pdm09 virus and CD3600 inhibits SARS-CoV-2 replication in hamster lungs when DIGs are administrated to lungs one day before viral challenge. To further investigate the gene delivery vector in the respiratory tract, a peptidic TAT2-P1&LAH4, which can package genes to form small spherical nanoparticles with high endosomal escape ability, is demonstrated to dramatically increase gene expression in the lung airway. TAT2-P1&LAH4, with the dual-functional TAT2-P1 (gene-delivery and antiviral), can deliver CD3600 to significantly inhibit the replication of Delta and Omicron SARS-CoV-2 in hamster lungs. This peptide-based nanoparticle system can effectively transfect genes in lungs and deliver DIGs to inhibit SARS-CoV-2 variants and influenza virus in vivo, which provides the new insight into the drug delivery system for gene therapy against respiratory viruses.

## Introduction

Clinical studies showed that early treatment or prophylactic treatment is more effective for controlling the viral respiratory diseases such as influenza. Apart from the therapeutic antivirals being developed, the prophylactic antivirals play important roles in preventing transmission, especially in outbreak settings. Vaccination is currently the most effective strategy in preventing viral infection, but vaccine effectiveness was virus strain dependent and varied from 10–60% protection for influenza.^1^ Vaccine effectiveness of SARS-CoV-2 has been affected by the variants.^2,3^ The onset of effective vaccine protection requires at least two weeks to generate sufficient titers of neutralizing antibodies. The neuraminidase inhibitors (zanamivir and oseltamivir) against influenza viruses are approved for prophylaxis, but both need to be administrated daily and there are concerns about drug resistance if these neuraminidase inhibitors are frequently used for prophylaxis. Antivirals directly targeting the virus, such as remdesivir, only showed benefits for patients with the mild or moderate symptoms,^4–6^ which implicates that early treatment or prophylactic treatment should be considered for COVID-19 cases. Thus, it is necessary to develop prophylactic antivirals with new antiviral mechanism and broad-spectrum antiviral efficacy for emerging influenza virus and coronavirus, especially for people with risk factors of susceptibility and/or severe diseases.

Defective interfering genes (DIGs) are viral genes with internal deletion.^7^ Influenza DIGs could inhibit the replication of the cognate full-length viral RNA and the antiviral activity is affected by DIG lengths and the origin of DIGs.^8–12^ The identification of sub-genomic RNAs of coronaviruses implicated that coronaviruses could generate defective genes during viral replication. However, it is not clear which DIG works best for inhibiting influenza virus and coronavirus in vitro and in vivo. We have previously found that influenza defective interfering genes (DIG-3) delivered by TAT-P1 in mouse lungs could protect mice from influenza A virus infection.^12^ DIG antiviral activity in lungs was related to the antiviral activity of DIGs and the transfection efficiency of DIGs in the lung airway. The gene transfection mediated by HIV TAT peptide is mainly through the endocytosis, including clathrin-mediated endocytosis,^13^ caveolae/lipid-raft-mediated endocytosis,^14^ micropinocytosis,^15^ and endocytosis independent pathways.^16^ In the airway and lungs, the passive cellular uptakes of nanoparticles are mainly mediated through endocytosis pathways and affected by the sizes of nanoparticles because of the barrier effect from airway mucus.^17–19^ One feasible way to increase the uptake efficiency in the airway and lung is to optimize the nanoparticle size to reduce the barrier effect of mucus so as to overcome the limit set by the average pore sizes (100–200 nm) in the airway mucus.^18,20,21^ Although nanoparticle-based drug delivery systems have been studied for decades, a simple gene delivery system with a reproducible and scalable product is needed for gene therapy in the lung airway.

In this study, we first investigated that the defective interfering gene (CD3600) designed from SARS-CoV-2 significantly inhibited the replication of SARS-CoV-2 variants in human HK-2 cells, which indicated that DIG from non-segmented RNA virus could significantly inhibit SARS-CoV-2 replication. We also demonstrated that a defective interfering influenza PA gene (PAD4) showed the best antiviral activity in human A549 cells when compared with twelve other DIGs from polymerase genes of segmented-RNA influenza virus. Treatment of mice with the combination of three defective interfering gene DIG-4 (including PAD4, PB1D3, and PB2D3) conferred the best protection against A(H1N1)pdm09 virus when compared with that of mice treated by PAD4 or DIG-3. A single dose of peptidic nanoparticle TAT2-P1/DIG-4 could efficiently protect 90% mice when TAT2-P1/DIG-4 was given to mice at 3-day before A(H1N1)pdm09 challenge. A single dose of peptidic nanoparticle TAT2-P1/CD3600 could inhibit SARS-CoV-2 replication in hamsters when CD3600 was given to hamster lungs at 1-day before viral challenge. To investigate the delivery vector for gene therapy in lungs, we further developed a peptidic TAT2-P1&LAH4, which significantly increased luciferase expression in lungs, could deliver CD3600 to broadly inhibit SARS-CoV-2 replication of Delta and Omicron variants in hamster lungs. Results from these experiments demonstrated that peptidic nanoparticle DIGs could provide pre-exposure protection on animals infected with influenza virus or SARS-CoV-2. Because of the low possibility of DIGs inducing drug-resistant virus^22,23^ and less metabolic toxicity of peptide, peptidic DIG nanoparticles can circumvent the drug-resistance problems with broad-spectrum antiviral activities against SARS-CoV-2 and influenza virus.

## Results

### Coronavirus DIGs inhibited the replication of SARS-CoV-2 in human HK-2 cells

**Fig. 1:**
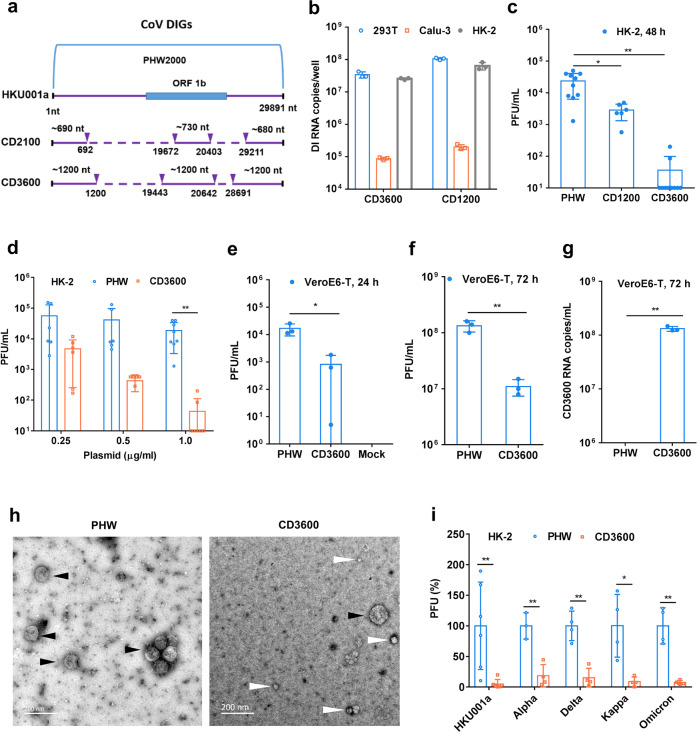
SARS-CoV-2 DIG construction, expression and antiviral activity in cells. **a** Construction of SARS-CoV-2 DIG (CD2100 and CD3600). Dotted lines show the internal deletion in the wild-type HKU001a (SARS-CoV-2). The three solid-line sequences linked together were inserted into phw2000 vector to generate CD2100 and CD3600. **b** DIG RNA expression in 293 T, Calu-3 and HK-2 cells (*n* = 3). After 24 h plasmid transfection, RNA expression in cells was measured by RT-qPCR. **c** The antiviral activity of DIG (1.0 µg ml^−1^) in HK-2 cells (*n* > 6). SARS-CoV-2 (B.1.1.63) was added to DIG-transfected cells for viral replication. At 48 h post infection, the viral titers in cell supernatants were measured by plaque assay. **d** Does-dependent antiviral activity of CD3600. CD3600 or PHW (1.0 µg, 0.5 µg, or 0.25 µg ml^−1^) were transfected to HK-2 cells one day before viral infection. Viral titers in cell supernatants were measured at 48 h post infection. **e**, **f** Antiviral activity of CD3600 during multi-cycle viral replication after viral passage (*n* = 3). The supernatant viruses (50 PFU) collected from HK-2 cells, transfected with PHW or CD3600 and then infected with SARS-CoV-2, were passaged in VeroE6-TMPRSS2 (VeroE6-T) cells. The viral titers in supernatants were determined at 24 h and 72 h post infection. **g** Supernatant CD3600 RNA copies of PHW- and CD3600-treated virus (*n* = 3) were determined at 72 h post infection. **h** Representative images of viral particles of PHW- or CD3600-treated SARS-CoV-2. Viruses (50 PFU of PHW or CD3600) were cultured in VeroE6-T and then were harvested at 72 h post infection for RT-qPCR and TEM assay. White arrows indicated the small spherical particles. Black arrows indicated the wild-type virus. Scale bars, 200 nm. **i** The broad-spectrum antiviral activity of CD3600 (0.5 µg ml^−1^) against SARS-CoV-2 variants in HK-2 cells (*n* = 4). PFU (%) was the plaque number of CD3600-treated virus normalized to the plaque number of PHW-treated virus. Data were presented as mean ± SD of independent biological samples. * indicates *P* < 0.05 and ** indicates *P* < 0.01. *P* values were calculated by the two-tailed Student’s *t* test

To identify DIGs against SARS-CoV-2, according to the possible packaging sequences of coronaviruses including 5’-end, sequences in ORF1b (nsp15), and 3’-end sequences,^24–27^ we constructed coronavirus DIGs (CD2100 and CD3600) which included the 5’-end sequences, internal sequences in ORF 1b and the 3’-end sequences of SARS-CoV-2 (Fig. 1a and Supplementary Table 1). We first tested the gene expression of DIGs transfected into human cell lines, including 293 T, Calu-3 and HK-2. High RNA levels of CD2100 and CD3600 were demonstrated in transfected HK-2 cell line, similar to that of RNA expression in 293 T cells (Fig. 1b). Thus, we selected HK-2^28^ for the antiviral assay of CD2100 and CD3600 against SARS-CoV-2. After DIG transfection in HK-2 cells, SARS-CoV-2 was added to cells for infection. CD3600 and CD2100 significantly inhibited SARS-CoV-2 replication when compared with empty vector PHW (Fig. 1c) without obvious cytotoxicity (Supplementary Fig. 1). CD3600 showed significantly better antiviral activity than that of CD2100. To further confirm the antiviral efficacy of CD3600 in cells, we showed that CD3600 significantly inhibited SARS-CoV-2 replication in the dose-dependent manner when compared with PHW (Fig. 1d).

In order to test if coronavirus DIG could exert antiviral activity in multiple growth cycles which can contribute to the expanded antiviral activity of DIG, we demonstrated that virus in supernatants of cells treated with CD3600 showed significantly less viral replication at 24 h and 72 h post infection during viral passages in VeroE6/TMPRSS2 cells when compared with non-DIG empty vector (PHW) control (Fig. 1e, f). These indicated that the transfected CD3600 could exert extended activity against SARS-CoV-2 replication even during viral passages in multiple growth cycles. To further investigate the presence of CD3600 defective virus in passaged supernatants in VeroE6/TMPRSS2 cells, we cultured the same PFU of PHW-treated virus and CD3600-treated virus and collected the supernatants at 72 h post infection for ultracentrifugation to recover the virus for transmission electron microscopy (TEM) assay (Fig. 1g and Supplementary Fig. 2). CD3600-treated cell culture supernatants showed more small spherical particles (<40 nm) when compared with PHW-treated cell culture supernatants (100–150 nm), which indicated that treatment with CD3600 could led to the packaging of smaller particles with less plaque form unit (Fig. 1f). As expected, RT-qPCR showed high CD3600 RNA copies in the supernatants (Fig. 1g) of CD3600-treated virus when compared with PHW-treated virus. These results indicated that CD3600 could be packaged to show the extended antiviral activity in the subsequent virus passage. Moreover, we demonstrated the broad antiviral activity of CD3600 against SARS-CoV-2 variants (Fig. 1i), which was consistent with the antiviral mechanism of DIG against virus by consuming the packaging components of viral replication to competitively inhibit wild-type viral RNA packaging without targeting a specific viral protein. In conclusion, these results indicated that CoV-DIG with the 5’-end, internal sequence in ORF1b, and 3’-end of SARS-CoV-2 could broadly inhibit SARS-CoV-2 replication by maintaining its self-sustaining DIG production in multiple growth cycles.

### The optimal DIG against influenza virus in A549 cells and in mice

**Fig. 2:**
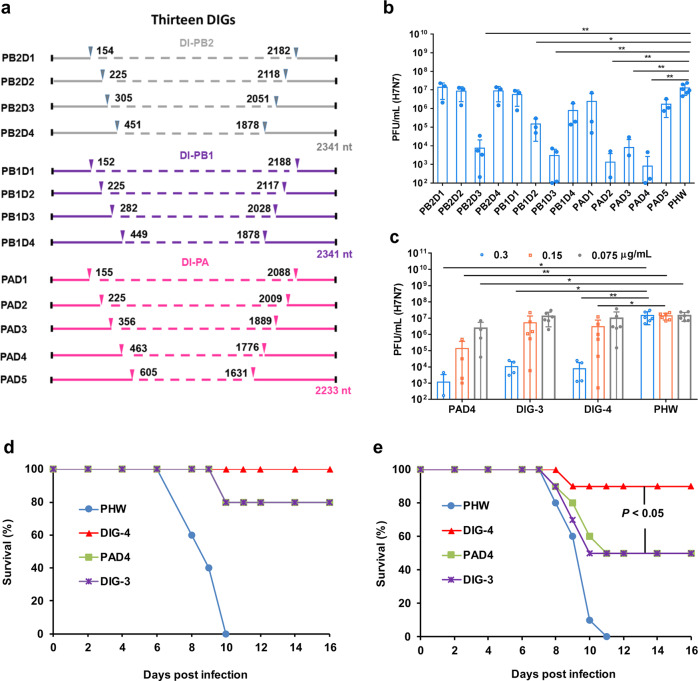
The design of diverse lengths of influenza DIGs and the antiviral efficiency of DIGs. **a** Construction of defective interfering genes (DIGs). The diverse defective interfering (DI) genes of DI-PA, DI-PB1 and DI-PB2 with internal deletion were generated by fusion PCR. Dotted lines show the internal deletion in wild-type viral polymerase genes. The solid-line sequences of shortened viral polymerase gene PA, PB1 and PB2 were inserted into phw2000 vector, respectively. **b** The antiviral activity of single DIG against A(H7N7) virus in A549 cells. **c** The dose-dependent antiviral efficiency of single PAD4 and combinational DIG-3 (including PAD3, PB2D3, and PB1D3) and DIG-4 (including PAD4, PB2D3, and PB1D3). The plasmid DIG or empty vector (PHW) with indicated concentrations were transfected into A549 cells and then cells were infected by H7N7 virus at 24 h post transfection. Viral titers in cell supernatants were measured by plaque assay at 48 h post infection. Data were presented as mean ± SD of at least three independent experiments. * indicates *P* < 0.05 and ** indicates *P* < 0.01. *P* values were calculated by the two-tailed Student’s *t* test. **d** The protection of two doses of DIG on A(H1N1)-infected mice. DIG-4 (5 µg/mouse, *n* = 5), PAD4 (5 µg/mouse, *n* = 5), DIG-3 (5 µg/mouse, *n* = 5), or empty vector (PHW, 5 µg/mouse, *n* = 5) packaged by TAT-P1 were intratracheally inoculated into mice at 48 h and 24 h before viral challenge. **e** The protection of one dose DIG on A(H1N1)-infected mice. DIG-4 (5 µg/mouse, *n* = 10), PAD4 (5 µg/mouse, *n* = 10), DIG-3 (5 µg/mouse, *n* = 10), or PHW (5 µg/mouse, *n* = 10) packaged by TAT-P1 were intratracheally inoculated into mice at 24 h before viral challenge. Survivals were generated from 10 mice in each group. *P* values were calculated by Gehan–Breslow–Wilcoxon test

To identify the potent antiviral DIG against influenza virus, based on our previous DIGs (PAD3, PB1D3 and PB2D3),^12^ we here designed and constructed ten new DIGs with diverse gene lengths at 3’ and 5’ ends of polymerase genes ranging from 150 nt to 600 nt (Fig. 2a, Supplementary Table 2 and Supplementary Table 3). Each DIG was transfected into A549 cells and then cells were infected with H7N7 virus at 24 h post transfection. We used H7N7 virus which did not require trypsin for virus culture in A549 cells (Fig. 2b). The PAD4, which consists of 450 nt at 3’ and 5’ end of PA gene segment, exhibited the most potent antiviral activity with 4 log reduction of viral load, which was 4 to 10 folds lower than that of PAD3, PB1D3 and PB2D3 used in the previous study.^12^ The PB2D3, PB1D3, PAD2, and PAD3 showed potent antiviral activity with 2–3 log inhibition of viral replication. The PB2D2, PB2D4, PB1D2, PB1D4, and PAD5 showed weak antiviral activity (Fig. 2b). The PB2D1, PB1D1, and PAD1 with ~150 nt at 3’ and 5’ ends did not show significant antiviral activity, which is consistent with the fact that the polymerase genes need at least 150 nt at 5’ end for viral gene packaging, as demonstrated by previous studies.^9,29^ These data indicated that PAD4 with 450 nt at 3’ and 5’ ends showed the best antiviral activity. The PB2D3 with 300 nt and PB1D3 with 300 nt at 3’ and 5’ ends showed better antiviral activity than those with shorter or longer DIGs, respectively. Next, we further tested and compared the antiviral activity of single PAD4 with two combinational DIGs (Fig. 2c) in different concentrations. The PAD4 alone showed more potent antiviral activity with > 5-fold lower viral load than that of DIG-3 (the combination of PAD3, PB1D3, and PB2D3) or the combination of DIG-4 (PAD4, PB1D3, and PB2D3) in a dose-dependent manner.

Considering multiple DIGs having higher likelihood to interfere with multiple cognate full-length genes in vivo, we selected DIG-4, single PAD4, and DIG-3 to evaluate the antiviral efficacy in mice. Theoretically, three DIGs can generate seven types of defective interfering influenza viruses, which might have more likelihood to inhibit wild-type virus replication than that of single DIG in mouse lungs. To transfect DIG in vivo, DIGs packaged by TAT-P1 were intratracheally inoculated to mice. Two doses of DIG-4, single PAD4, or DIG-3, given at 48 h and 24 h prior to A(H1N1)pdm09 infection, could protect 100%, 80%, and 80% mice from lethal challenge by A(H1N1)pdm09 virus, respectively (Fig. 2d), which was significantly better than that of empty plasmid PHW (0%). For 1-dose regimen given intratracheally at 24 h prior to infection (Fig. 2e), the survival rate of mice receiving DIG-4 (90%) was significantly higher than those receiving single PAD4 (50%) or DIG-3 (50%). DIG-4 could significantly reduce body weight loss of mice when compared with PHW (Supplementary Fig. 3). The survival data indicated that combinational DIG-4 can provide better protection than the single PAD4 or DIG-3 in mice.

### TAT2-P1 showed better gene delivery efficiency in lung tissues

**Fig. 3:**
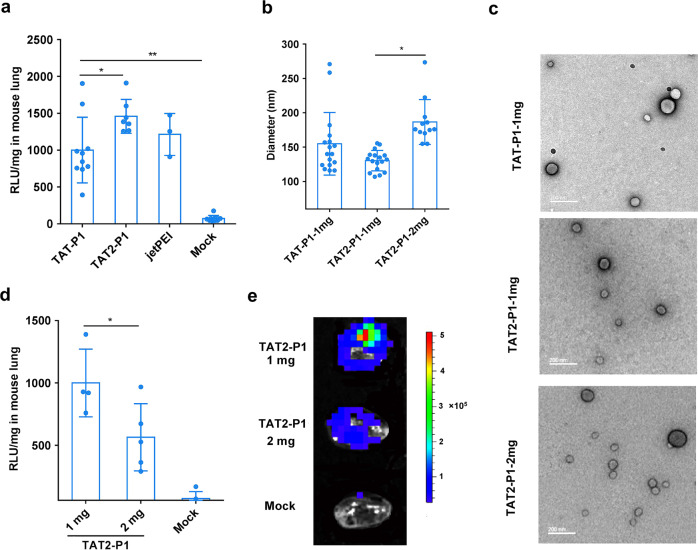
Gene transfection efficiency of TAT2-P1 in vivo. **a** The transfection efficiency of TAT-P1, TAT2-P1, and in vivo jetPEI in mouse lungs. The pCMV-Luc was packaged by the indicated vectors with the weight ratio (4:1) of peptide:DNA. The luciferase expression in mouse lungs was measured at 24 h post transfection. Luciferase expression in mouse lungs was normalized to TAT-P1 (1000). Mock means mice treated with 5% glucose without pCMV-Luc. * indicates *P* < 0.05 and ** indicates *P* < 0. **b** Hydrodynamic diameter of nanoparticles of plasmid DNA packaged by indicated vectors. Sizes were measured by DynaPro Plate Reader. Data were presented as mean ± SD of six independent experiments. **c** The representative pictures of transmission electron micrographs (TEM) of nanoparticles. Peptides (1 mg ml^−1^ or 2 mg ml^−1^ for TAT2-P1-2mg) were mixed with equal volume of plasmid DNA (0.25 mg ml^−1^ or 0.5 mg ml^−1^) for measuring hydrodynamic diameter or for TEM negative staining. Scale bars, 200 nm. **d** The expression of luciferase in mouse lungs transfected with nanoparticle TAT2-P1/pCMV-Luc (1 mg ml^−1^/0.25 mg ml^−1^) and TAT2-P1/pCMV-Luc (2 mg ml^−1^/0.5 mg ml^−1^), respectively. Data were presented as mean ± SD of more than four mice in each group. **e** Representative image of In Vivo Imaging System showed decreased luciferase expression of pCMV-Luc packaged by 2 mg ml^−1^ of TAT2-P1 (TAT2-P1-2mg) in mouse lungs. The same amount of TAT2-P1/pCMV-Luc was inoculated to corresponding mouse lungs at 24 h before measuring luciferase expression. Mock, mouse lungs inoculated with TAT2-P1. * indicates *P* < 0.05. *P* values were calculated by the two-tailed Student’s *t* test

In order to show better antiviral activity of DIG in vivo, we tried to identify a TAT-peptide based vector with better transfection efficiency in vivo. Previous studies indicated that TAT2 (RKKRRQRRR) is a shorter form of TAT which had better transduction efficiency than that of TAT (YGRKKRRQRRR) in vitro.^30,31^ Therefore, we constructed a TAT2-P1 vector for evaluating the transfection efficiency in vitro when compared with TAT-P1 (Supplementary Table 4). DNA binding ability of these peptide-based vectors was determined using gel retardation assay (Supplementary Fig. 4a). When the weight ratios of peptide to plasmid DNA were more than 2, plasmid DNA could be completely packaged by these vectors. The in vitro transfection results indicated that TAT-P1 and TAT2-P1 showed similar transfection in 293 T cells (Supplementary Fig. 4b). Next, to assess the DNA transfection efficiency of these vectors in vivo, pCMV-Luc packaged by TAT-P1, TAT2-P1 or in vivo jetPEI was intratracheally inoculated into mice, and the luciferase was measured at 24 h post transfection. TAT2-P1 showed significantly better transfection efficiency in mouse lungs than that of TAT-P1 (Fig. 3a) and TAT2-P1 caused less body weight loss than that of TAT-P1 in mice (Supplementary Fig. 5).

To identify the mechanism of better transfection efficiency of TAT2-P1 in vivo, we measured the particle sizes of these peptide/pCMV-Luc particles. The pCMV-Luc packaged by TAT2-P1 could form smaller particles than that of TAT-P1 (Fig. 3b). TEM pictures of these nanoparticles (Fig. 3c) showed that the particles formed by TAT2-P1 were uniform spherical nanoparticles, which could be beneficial to penetrate the airway mucosal barrier.^17,18^ The higher transfection efficiency of TAT2-P1 in mouse lungs should be related to the small particle sizes but not due to the transmission efficiency in cells because TAT2-P1 and TAT-P1 showed similar transfection efficiency in cells (Supplementary Fig. 4b). In order to directly confirm the particle size significantly affecting the gene delivery of TAT2-P1 in lungs, we made TAT2-P1/pCMV-Luc with different sizes by mixing TAT2-P1 with DNA in different concentrations but with the same amount of peptide/DNA and ratio (4:1). As shown in Fig. 3b, c, the particle size of TAT2-P1/pCMV-Luc (2 mg ml^−1^/0.5 mg ml^−1^) was bigger (~190 nm) than that (~130 nm) of TAT2-P1/pCMV-Luc (1 mg ml^−1^/0.25 mg ml^−1^). Furthermore, pCMV-Luc packaged by TAT2-P1 (1 mg ml^−1^) showed significantly higher transfection efficiency than that of pCMV-Luc packaged by TAT2-P1 (2 mg ml^−1^) in mouse lungs (Fig. 3d, e), but the transfection efficiency of TAT2-P1 (2 mg ml^−1^) was similar as TAT2-P1 (1 mg ml^−1^) in 293 T cells (Supplementary Fig. 6). These data indicated that the higher transfection efficiency of plasmid DNA packaged by TAT2-P1 (1 mg ml^−1^) than that of TAT2-P1 (2 mg ml^−1^) in mouse lungs was not due to the difference of DNA package efficiency, but due to the small nanoparticles. Collectively, these results demonstrated that TAT2-P1/DNA caused less body weight loss of mice and could form uniform spherical nanoparticles less than 140 nm, which would penetrate the barrier of the average pore sizes (100–200 nm) in airway mucus so as to increase DNA uptakes for high gene expression in the lung airway.

### Antiviral activity of DIGs against influenza virus and SARS-CoV-2

**Fig. 4:**
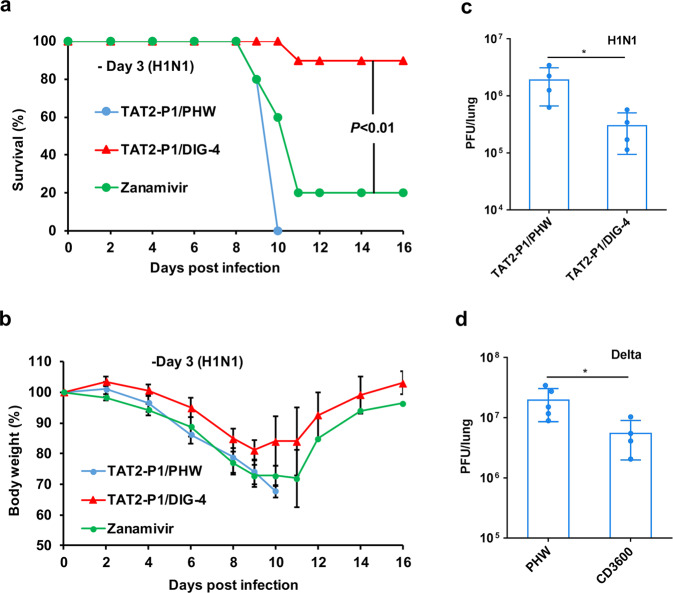
One dose of TAT2-P1/DIG showed rapid-onset prophylactic protection on infected animals. **a** The protective efficacy of antivirals administrated to mouse lungs 3-day before A(H1N1)pdm09 virus challenge. TAT2-P1/DIG-4 (20 µg/5 µg, *n* = 10), zanamivir (40 µg, *n* = 5), or TAT2-P1/PHW (20 µg/5 µg, *n* = 5) were intratracheally inoculated into corresponding mice 3-day before A(H1N1)pdm09 virus challenge. **b** The body weight changes of infected mice corresponding to (**a**). **c** Viral loads in H1N1-infected mouse lungs treated with TAT2-P1/PHW or TAT2-P1/DIG-4 at 3-day before virus challenge. Viral titers were measured at 2-day post infection by plaque assay. **d** CD3600 could inhibit delta SARS-CoV-2 replication in hamster lungs. TAT2-P1/CD3600 (50 µg/12.5 µg, CD3600, *n* = 4) was inoculated to hamster lungs at day 1 before SARS-CoV-2 (Delta) challenge. TAT2-P1/PHW (PHW, *n* = 5) was given to hamster lungs at day 1 before viral challenges. Lung tissues were collected at 2-day post infection for measuring viral loads by plaque assay. Data were presented as mean ± SD of 4–5 hamsters in each group. * indicates *P* < 0.05, calculated by the two-tailed Student’s *t* test

To investigate prophylactic protection of DIG in vivo, a single dose of TAT2-P1/DIG-4 was given to influenza-infected mice 3-day before viral challenge. The survival (90%) of mice treated with single dose of TAT2-P1/DIG-4 was higher than that of mice treated with a single dose of zanamivir (20%) or TAT2-P1/PHW (0%, negative control) (Fig. 4a). Significantly less body weight loss was observed in TAT2-P1/DIG-4 treated mice from day 2 to day 8 when compared with TAT2-P1/PHW group (Fig. 4b). To test the effectiveness of zanamivir, a single dose of zanamivir administrated to mice one day before viral challenge could protect 90% mice (Supplementary Fig. 7), which was consistent with the half-life time of zanamivir.^32^ The effective antiviral activity of TAT2-P1/DIG-4 was further demonstrated by the reduced viral loads in mouse lungs when compared with viral loads in mice treated with TAT2-P1/PHW (Fig. 4c). These results provided the evidence of using TAT2-P1/DIG as a prophylactic antiviral against influenza virus.

To evaluate the in vivo antiviral activity of CD3600 against SARS-CoV-2, TAT2-P1/CD3600 was inoculated into hamster lungs at 1-day before SARS-CoV-2 inoculation. Because of the non-lethal model of hamsters for SARS-CoV-2 infection and the peak viral titer emerged at 2-day post infection in hamster lungs,^33,34^ viral titers in hamster lungs were measured by plaque assay at 2-day post infection. The result showed that CD3600 inhibited SARS-CoV-2 replication in hamster lungs when compared with PHW (Fig. 4d). In conclusion, these in vivo data showed that TAT2-P1 could deliver DIGs in the lung airway to provide prophylactic protection on mice against influenza A virus and on hamsters against SARS-CoV-2 replication.

### TAT2-P1&LAH4 showed high-efficiency gene transfection in lung tissues

**Fig. 5:**
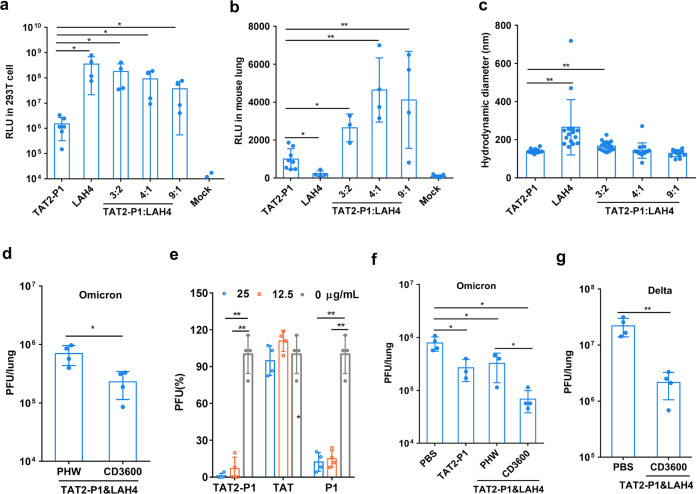
TAT2-P1&LAH4 enhanced the gene expression and inhibited SARS-CoV-2 variants in vivo. **a** The luciferase expression in 293 T cells. The pCMV-Luc was packaged by the indicated vectors (TAT2-P1, LAH4, and TAT2-P1:LAH4 = 3: 2, 4:1 or 9:1) and was transfected into 293 T cells (*n* = 4). **b** The luciferase expression in mouse lungs (*n* = 4). The pCMV-Luc was packaged by the indicated vectors and was inoculated to mouse lungs. The expression of luciferase in mouse lungs was detected at 24 h post transfection. **c** Peptidic nanoparticle sizes of plasmids packaged by the indicated vectors (*n* = 4). * indicates *P* < 0.05 and ** indicates *P* < 0.01. **d** TAT2-P1&LAH4 could deliver CD3600 to significantly inhibit Omicron (*n* = 3) variant replication in hamster lungs. One dose of CD3600 was intratracheally inoculated to hamster lungs at 1-day before viral challenge. Viral loads in hamster lungs were measured at 2-day post infection. **e** TAT2-P1 significantly inhibit SARS-CoV-2 infection (*n* = 4) in VeroE6 cells. SARS-CoV-2 treated by indicated peptides for plaque reduction assay. PFU (%) was the plaque number of peptide-treated virus normalized to the plaque number of untreated virus. ** indicates *P* < 0.01. **f** Two doses of PBS (*n* = 4), TAT2-P1 (*n* = 3), TAT2-P1&LAH4 with CD3600 (*n* = 4) or PHW (*n* = 3) were given to hamster lungs at 1-day before and 8 h after Omicron SARS-CoV-2 infection. **g** PBS (*n* = 4) or TAT2-P1&LAH4 with CD3600 (*n* = 4) were given to hamster lungs at 1-day before and 8 h after Delta SARS-CoV-2 infection. Viral loads were measured at 2-day post infection. * indicates *P* < 0.05 and ** indicates *P* < 0.01*. P* values were calculated by the two*-*tailed Student’s *t* test

To further investigate peptide-based vectors for gene delivery in the lung airway, we demonstrated that peptide LAH4^35^ showed significantly higher transfection efficiency than TAT2-P1 in cells (Fig. 5a) with TC_50_ higher than 125 μg ml^−1^ (Supplementary Fig. 8), but significantly lower transfection efficiency than TAT2-P1 in lung tissues (Fig. 5b). The high transfection efficiency in cells indicated that the high endosomal escape ability of LAH4-packaged plasmids demonstrated by previous study.^35^ However, the low transfection efficiency in lung tissues might be due to the big size of LAH4-nanoparticles (Fig. 5c) which were not spherical in TEM pictures (Supplementary Fig. 9). Because the size and the shape could be the barrier to pass the mucosal layer in lung tissues,^17,18,36^ our hypothesis was that we might increase the transfection efficiency by using TAT2-P1 plus LAH4 which could form the small spherical nanoparticles with high endosomal escape ability. When the ratio of TAT2-P1:LAH4 was 4:1 (Fig. 5c and Supplementary Fig. 9), TAT2-P1&LAH4-pCMV could form spherical nanoparticles (~140 nm). The transfection efficiency (4:1) was significantly higher than that of TAT2-P1 in cells (Fig. 5a) and the TAT2-P1&LAH4 nanoparticles could be stable in room temperature for more than 72 h (Supplementary Fig. 10). Importantly, TAT2-P1&LAH4 showed significantly higher transfection efficacy in mouse lungs than that of TAT2-P1 or LAH4 only (Fig. 5b). These results further demonstrated that small nanoparticle sizes (TAT2-P1&LAH4) were important for penetrating airway mucus to increase gene expression in lungs.

### TAT2-P1&LAH4/CD3600 inhibited SARS-CoV-2 variants in hamsters

We first demonstrated that TAT2-P1&LAH4/CD3600 inhibited the replication of SARS-CoV-2 (Omicron) in hamster lungs when one dose was given to hamsters at one day before viral challenge (Fig. 4d). Our previous study showed that defensin derived peptide P9R could inhibit SARS-CoV-2.^37^ P1 is derived from P9R (Supplementary Table 4). We further demonstrated that P1 and TAT2-P1 significantly inhibited SARS-CoV-2 replication (Fig. 5e). When two doses of TAT2-P1&LAH4/CD3600 were given to hamster lungs at 1-day before and 8-hour after SARS-CoV-2 challenge, TAT2-P1&LAH4/CD3600 could more effectively inhibit SARS-CoV-2 Omicron replication in hamster lungs (Fig. 5f). TAT2-P1&LAH4-PHW also inhibited the replication of SARS-CoV-2 (Fig. 5g), which could be attributed to the antiviral activity of TAT2-P1 against SARS-CoV-2 (Fig. 5e, f). The dual function of TAT2-P1 (gene-delivery and antiviral) conferred TAT2-P1&LAH4/CD3600 to show more potent antiviral activity against SARS-CoV-2 in vivo. Moreover, we demonstrated that two doses of TAT2-P1&LAH4/CD3600 significantly inhibited the replication of Delta SARS-CoV-2 in hamster lungs (Fig. 5f). The NP staining further indicated that CD3600 could reduce Omicron and Delta variant replication (Supplementary Fig. 11) in hamster lungs. These results indicated that peptidic TAT2-P1&LAH4 nanoparticles with the advantages of small spherical particles and high efficiency of endosomal escape ability could effectively penetrate airway mucus to express gene in the lung airway and deliver DIG to significantly inhibit the replication of SARS-CoV-2 variants in hamster lungs.

## Discussion

In this study, we designed new defective interfering genes (DIGs) of SARS-CoV-2 and influenza A virus and investigated the antiviral activity of DIGs against SARS-CoV-2 variants and influenza A virus. Peptidic DIG nanoparticles with the high efficiency of gene delivery in the lung airway could significantly inhibit SARS-CoV-2 variants and influenza A virus in animal models. With the development of high-efficient gene delivery vectors, DIGs can serve as broad-spectrum antivirals with high barrier to drug resistance.^22^

For respiratory virus diseases, vaccination is the most effective strategy for prevention. Short of vaccination, the early initiation of antiviral treatment is important for better outcome in patients, which is clearly shown in COVID-19 and influenza. Currently, prophylactic drugs for pre-exposure and post-exposure of influenza virus are recommended by WHO for daily administration for 10 days despite the concern of drug resistance. No widely available and effective drug is yet recommended for the prophylaxis of coronavirus.^38,39^ Thus, it is important to identify antivirals with new antiviral mechanism for pre- or post-exposure use, which should have the characteristics of rapid-onset and low possibility to induce drug resistant virus. For influenza A virus, one dose of DIG-4 or zanamivir could show prophylactic protection on infected mice when given one day prior to viral challenge. However, one dose of zanamivir could not provide the same protection as DIG-4 for three-day prophylactic protection in our experiments, which indicated that DIG-4 could have the long-term prophylactic antiviral activity against influenza virus.

SARS-CoV-2 vaccines have been found to be promising in controlling COVID-19 in many countries. Due to vaccine hesitancy and variants of concern, antivirals for SARS-CoV-2 are also needed as an alternative approach for the better control of SARS-CoV-2 infection and reduction of mortality. Recently, a therapeutic interfering particle showed promising antiviral activity against SARS-CoV-2,^22^ which used the lipid nanoparticles as the RNA delivery vector with different design of interfering genome when compared with our study using peptide-DIG nanoparticles. In this study, TAT2-P1&LAH4/CD3600 showed broad activity against SARS-CoV-2 variants in hamsters. The high transfection efficiency of TAT2-P1&LAH4 in the lung airway could be attributed to the small nanoparticle sizes adjusted by TAT2-P1 for better penetration in the airway mucus and the high endosomal escape ability adjusted by LAH4 for gene release. The development of peptide-based gene vectors, with less metabolic toxicity, can provide a promising system for the gene therapy in the lung airway. The toxicity testing showed that peptide-DIG nanoparticles did not show obvious toxicity in vitro (Supplementary Fig. 1) and could slightly reduce the body weight after day 1 inoculation and then the body weight could recover after day 3 inoculation (Supplementary Fig. 5). Further studies to discover peptide vectors causing less or no body weight loss and in vivo safety studies are valuable.

The shorter CD2100 showed less activity than that of CD3600 against SARS-CoV-2, and PAD4 showed the most potent activity against influenza A virus in vitro. These results indicated that the length of DIGs can determine the effectiveness of competitively inhibiting wild-type viral RNA replication.^40^ Apart from optimizing gene delivery system in the lung airway, further studies on DIGs might identify DIGs with more potent activity against SARS-CoV-2. In addition, the small spherical particles in the supernatants of CD3600-treated virus could show inhibition on viral replication in multiple viral growth cycles, which indicated that the small spherical particles could be virus-like particles with infectious activity and might have immunogenic activity in vivo. Further studies are warranted to investigate the potential possibility against SARS-CoV-2.

Previous studies showed that influenza defective interfering virus did not rely on interferon response to protect influenza challenged mice and could protect elderly mice.^41,42^ Also, the antiviral activity of influenza DIG was not relying on DIG protein expression.^8^ The rapid-onset of prophylactic antiviral activity of peptide-DIG nanoparticles against influenza virus in mice and SARS-CoV-2 in hamsters also implicated that the antiviral activity of DIG relies on the competitively inhibition on wild-type viral replication. The broad-spectrum antiviral activities of influenza DIG against different influenza A viruses and CD3600 against SARS-CoV-2 variants suggest that antiviral DIG is less prone to develop viral resistance.^8,22,23^ These peptide-DIG nanoparticles can be a potential candidate for providing the rapid-onset prophylactic protection with high barriers to drug resistance against influenza A virus and SARS-CoV-2 variants.

## Material and methods

### Animal experiments

Animal experiments in this study were evaluated and approved by the Committee on the Use of Live Animals in Teaching and Research of the University of Hong Kong.

### Cell and virus cultures

Madin Darby canine kidney (MDCK, CCL-34), A549 (CCL-185), 293 T (CRL-3216), VeroE6 (CRL-1586), Calu-3 (HTB-55),^43^ VeroE6-TMPRSS2 (VeroE6-T),and HK-2 (CRL-2190)^28^ cells from ATCC were cultured in Dulbecco minimal essential medium (MEM), DMEM or DMEM-F12K with 5–10 % fetal bovine serum, 100 μg ml^−1^ streptomycin and 100 IU ml^−1^ penicillin. Viruses used in this study included A/Hong Kong/415742Md/2009 (H1N1),^44^ A/Netherlands/219/2003 (H7N7),^45^ and SARS-CoV-2 variants.^46–48^ Influenza A viruses were cultured in MDCK cells, while SARS-CoV-2 variants were cultured in VeroE6 or VeroE6-TMPRSS2 cells,

### Coronavirus DIG construction and expression

According to previous studies which identified the possible packaging genes for coronavirus,^24,25,49^ we synthesized the 5’-end sequences (~700 and 1200 bp), the internal sequences in ORF1b and the 3’-end sequences of SARS-CoV-2 of HKU-001a (GenBank: MT230904.1, Supplementary Table 1). The synthesized genes were inserted into phw2000 vector to generate plasmid CD2100 and CD3600 for expressing defective genes of SARS-CoV-2. The constructed plasmid DIGs were verified by Sanger sequencing. The CD2100 and CD3600 expression in 293 T, A549 and HK-2 cells were measured by RT-qPCR after the plasmids were transfected into cells at 24 h post transfection. For determining DIG RNA expression, RNA samples were treated by DNase I (QIAGEN, Cat # 79254) and further purified by RNeasy Mini Kit (Qiagen, Cat # 74106). The RNA expression levels were detected by RT-qPCR with DIG primers (Supplementary Table 5).

### Influenza DIG construction

According to our previous DIGs having ~300 nt located at the 5’ and 3’ ends,^12^ we firstly constructed DIGs with ~150 nt and 450 nt located at the 5’ and 3’ ends (Supplementary Table 2 and Supplementary Table 3). Based on the antiviral activities in A549 cells, we further constructed DIGs with about 225 nt and 600 nt at the 3’ and 5’ ends. The wild-type A/WSN/1933 PB2, PB1 and PA genes were used as the templates to generate defective interfering PB2, PB1 and PA genes (DI-PB2, DI-PB1 and DI-PA). Short gene segments at the 5′ and 3’ ends of DIGs were amplified by specific primers (Supplementary Table 6 and Supplementary Table 7). The amplified short gene fragments were fused by fusion PCR^50^ to generate DI-PB2, DI-PB1 and DI-PA genes using two pairs of primers for each gene (Supplementary Table 6 and Supplementary Table 7). The fused DI-PB2, DI-PB1 and DI-PA genes (Supplementary Table 2 and Supplementary Table 3) were inserted into the phw2000 vector to generate plasmid DIGs of pDI-PA, pDI-PB1, and pDI-PB2, respectively. The constructed plasmid DIGs were verified by sequencing.

### In vitro DIG transfection and antiviral assay

To test the antiviral activity of DIGs in vitro, plasmid DIGs or empty vector phw2000 (PHW) were transfected into A549 or HK-2 cells by Lipofectamine 3000 reagent (Invitrogen, Cat # 1857483) or GeneJuice (Sigma, Cat # 70967). After 24 h transfection, cells transfection media were replaced by fresh DMEM with 0.005 MOI of A(H7N7) virus or 0.01 MOI of SARS-CoV-2 for infection in A549 (influenza) or HK-2 (SARS-CoV-2) cells. Supernatants viral culture were collected at 48 h post infection for measuring viral titers by plaque assay as what we described previously.^44^ The antiviral activity of DIG was generated by comparing the viral titers in supernatants of cells transfected with plasmid DIGs or PHW.

### Peptide synthesis

TAT-P1, TAT2-P1, LAH4, P1 and TAT were synthesized by the company of ChinaPeptide (Hefei, China) as shown in Supplementary Table 4. The purity of peptides was >90%. The mass and purity of peptide was verified by mass spectrometry and HPLC.

### Toxicity assay

To detect the cytotoxicity, the 50% cytotoxic concentration (CC_50_) of peptide was measured by MTT assay as what we described previously.^44^ The 293 T or HK-2 cells (3 × 10^4^ cells per well in DMEM) were seeded in 96-well cell culture plate supplemented with 10% FBS. After overnight culture, culture media were replaced with DMEM supplemented with different concentrations of peptides and 1% FBS. After 24 h culture at 37 °C, MTT (10 μl of 5 mg ml^−1^) was added to cells for incubation at 37 °C for 4 h, and then100 μl of 0.01 M HCl in 10% SDS was added to cells for further incubation at room temperature with shaking overnight. Finally, plates were read at OD_570_. Cells without peptides were used as the experiment control. The in vivo toxicity of nanoparticles was measured by testing the body weight change of mice intratracheally inoculated with 40 μl of TAT-P1/DNA (20 μg/5 μg) or TAT2-P1/DNA (20 μg/5 μg).

### Gel retardation assay

According to the previous study,^12^ peptide TAT-P1 and TAT2-P1 (1 mg ml^−1^, 0.5 mg ml^−1^ and 0.25 mg ml^−1^) and DNA (0.5 μg) were premixed at various weight ratios in 4 μl of H2O at room temperature for 15 mins. Samples were loaded into a 1.2% w/v agarose gel and gel electrophoresis was performed in TAE buffer at 100 V for 25 min and then the agarose gel was visualized by the ultraviolet (UV) illumination.

### Hydrodynamic sizes of nanoparticles

According to the previous study,^12^ peptides and DNA were mixed at various weight ratios. Peptides and plasmid DNA were prepared separately in H2O and then equal volumes of peptides and DNA were mixed together to give a final volume of 4 μl containing 0.5 μg of plasmid and DNA. After the incubation of the complexes at room temperature for 15 min, the 4 μl complexes were diluted to 50 μl by distilled water and then the particle sizes were measured by DynaPro^®^ Plate Reader (WYATT, USA).

### Transmission electron microscopy analysis

To determine virus or nanoparticle size and shape, virus or peptide/DNA nanoparticles were mixed with the weight ratio (4:1) of peptide:DNA for inoculation at room temperature for 30 min and then were applied to continuous carbon grids. The excess solution was removed. The grids were transferred into 4% uranyl acetate for 1 min incubation and then remove the solution. Finally, the grids were air-dried at room temperature.^37^ For each peptide/DNA nanoparticle, TEM images were taken from two independent experiments by FEI Tecnal G2-20 TEM.

### In vitro luminescence assay

Peptides (1 mg ml^−1^, 0.5 mg ml^−1^ and 0.25 mg ml^−1^) and plasmid DNA (0.5 μg) were mixed at various weight ratios in 4 μl of H2O. After incubation at room temperature for 15 min, 293 T cells in 24-well plate were transfected with the peptide/DNA complexes (4 μl in 400 μl DMEM media) including peptides and 0.1 μg of each pHW2000 plasmid encoding the NP, PA, PB1, PB2 and the mini-genome of pPoLI-fluc-RT (pLuciferase).^45^ Finally, the luminescence was measured by Luciferase assay system (Promega, Cat # E1910) at 24 h post transfection,.

### In vivo bioluminescence assay

Peptides and pCMV-Luc (ThermoFisher, Cat # RF233236) were prepared at weight ratios (4:1) of peptide:DNA with 20 μg peptides and 5 μg plasmid in 40 μl of H2O. After incubating the complexes for 15 min, the complexes were intratracheally inoculated to mice at 24 h before measuring the luciferase in mouse lungs. The in vivo jetPEI (Polyplus, Cat # 201-10 G) mixed with pCMV-Luc (0.7 μl + 5.0 μg) were used as a positive control. Mouse lungs inoculated with in vivo jetPEI or peptide only were collected as the negative control. To detect bioluminescence, lung tissues were homogenized and centrifuged at 16,000 × *g* for 10 min. The supernatants were used to measure the luciferase expression by Cypridina luciferase kit (ThermoFisher, Cat # 16168). The relative luciferase expression in lungs was normalized to 1 mg protein. For in vivo bioluminescence imaging, the substrate was added to lungs for taking images by IVIS^®^ Spectrum In Vivo Imaging System (PerkinElmer, USA).

### RT-qPCR assay

Real-time quantitative PCR (RT-qPCR) was performed as what we did previously.^44^ Extracted RNAs were transcribed to cDNA using primer Uni-12 or random primers by Strand cDNA synthesis Kit (Takara, Cat # 6210 A). The cDNA was then amplified using gene specific primers (Supplementary Table 5) for DI-PB2, DI-PA, DI-PB1, CD2100 and CD3600 using SYBR Green I Master (Takara). For quantitation, 10-fold serial dilutions of standard plasmid equivalent from 10^1^ to 10^6^ copies per reaction were used to generate the standard curve. RT-qPCR was performed using LightCycler^®^ 96 system (Roche, USA).

### Nucleocapsid (NP) immunofluorescence assay

Infected hamster lungs were collected at day 2 post infection. Tissues were processed for anti-nucleocapsid assay. Tissue slides were blocked by Sudan Black B and 1% BSA at room temperature for 1 h. Slides were incubated with rabbit IgG anti-NP (1:1000)^51^ at room temperature for overnight at 4 °C and then washed by PBS and then incubated with goat anti-rabbit IgG Alexa-488 (Life Technologies, Cat # A32731, 1:200) at room temperature for 30 min. Finally, slides were washed by PBS and incubated with DAPI for nucleus staining. Fluorescence images were taken by confocal microscope (LSM 900, Germany).

### In vivo antiviral assay

Antiviral analysis of nanoparticle DIG in vivo was carried out in BALB/c mice and hamsters.^43^ Female BALB/c mice (9–12 weeks) and female hamsters (4–6 weeks) were obtained from The University of Hong Kong Centre for Comparative Medicine Research. All experimental protocols followed the standard operating procedures of the approved biosafety level 2/3 animal facilities.^52^ For the influenza DIG experiments, we used the mouse adapted A(H1N1)pdm09 virus to challenge mice to investigate the DIG antiviral activity. To identify the prophylactic efficacy, mice were intratracheally inoculated with 40 μl of TAT-P1/DIG (20.0 μg/5.0 μg), TAT2-P1/DIG (20.0 μg/5.0 μg), or zanamivir (40.0 μg/mouse in PBS) at 1–3 days before viral challenge. After nanoparticle DIG inoculation, mice were intranasally inoculated with 3 LD_50_ of A(H1N1)pdm09 virus. Survivals and general conditions were monitored for 16 days or until death. For DIGs against SARS-CoV-2 variants including Delta and Omicron in hamsters, TAT2-P1/CD3600, TAT2-P1/PHW, TAT2-P1&LAH4/CD3600, TAT2-P1&LAH4/PHW (50.0 μg/12.5 μg), or TAT2-P1 (50 μg) were intratracheally inoculated to hamsters at 1-day before viral challenge (250 PFU) and intranasally inoculated to hamsters at 8 h post infection. Experimental animals were randomly allocated to each group. Viral loads in hamster lungs were tested at 2-day post infection.

## Supplementary information

Supplementary Figures and tables

## Data Availability

All data that support the conclusions of the study are available from the corresponding authors upon a reasonable request.
