# Supplementary material for: Peptidic defective interfering gene nanoparticles against Omicron, Delta SARS-CoV-2 variants and influenza A virus in vivo
Source: Signal Transduct Target Ther. 2022 Aug 3;7:266. doi: 10.1038/s41392-022-01138-0 (PMC9349215; doi:10.1038/s41392-022-01138-0)
Supplement: Supplementary file 1 — Supplementary Figures and tables [file 41392_2022_1138_MOESM1_ESM.docx]

Supplementary Materials for

**Peptidic defective interfering gene nanoparticles against Omicron, Delta SARS-CoV-2 variants and influenza A virus in vivo**

# Hanjun Zhao, Chuyuan Zhang, Hoiyan Lam, Xinjie Meng, Zheng Peng, Man Lung Yeung, Jasper Fuk-Woo Chan, Kelvin Kai-Wang To, Kwok-Yung Yuen

Correspondence to: hjzhao13@hku.hk; kyyuen@hku.hk

**This PDF file includes:**

Figures. S1 to S11

Tables S1 to S7


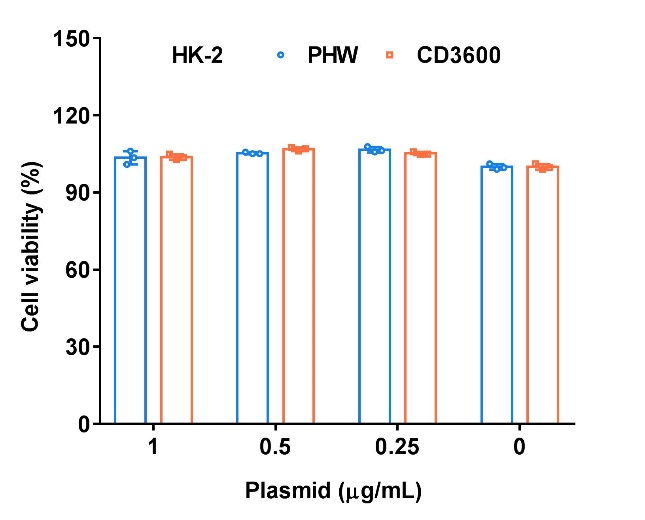


**Supplementary Fig. 1. Cytotoxicity of CD3600 transfected in HK-2 cells.** The indicated concentrations of CD3600 or PHW were transfected in HK-2 cells for 24 h. The cytotoxicity was measured by MTT assay. Cell viability (%) was the OD value of the transfected cells normalized to that of cells without transfection. Data were presented as mean ± SD of three biological samples.


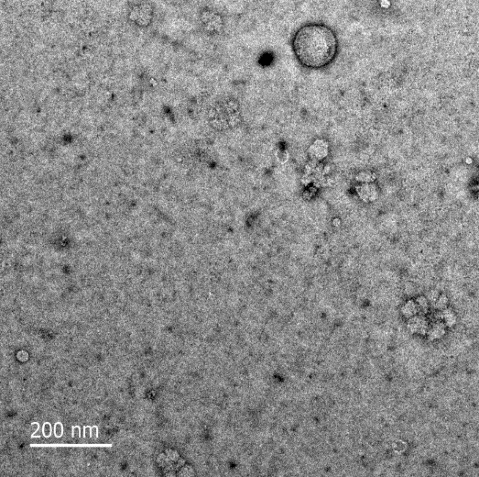

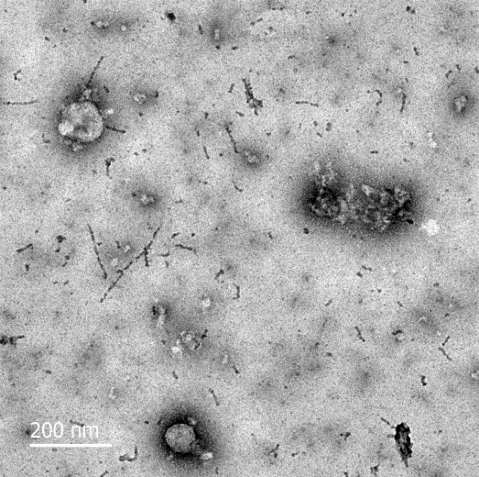


**PHW**

**CD3600**

**Mock**


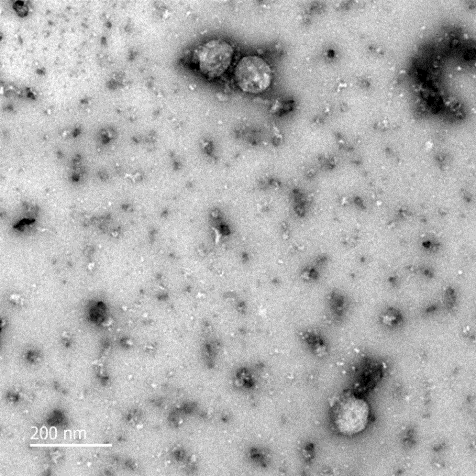


**Supplementary Fig. 2. Viral particles of SARS-CoV-2 treated by CD3600 or PHW.** CD3600-treated virus and PHW-treated virus (50 PFU) were cultured in Vero-E6 TMPRSS2 cells and supernatant viruses were collected at 3-day post infection for TEM assay. Representative images indicated that CD3600-treated virus showed more small spherical particles (< 40 nm) when compared with PHW-treated virus (100-150 nm). Wild-type virus without treatment (Mock) was the control. White arrows indicated the small spherical particles. Black arrows indicated the wild-type virus. Scale bar = 200 nm.

**a**

**b**

**Supplementary Fig. 3. Body weight changes of infected mice.** (**a**) Two doses of DIG-4 (5 µg/mouse), PAD4 (5 µg/mouse), DIG-3 (5 µg/mouse), or empty vector (PHW, 5 µg/mouse) packaged by TAT-P1 were intratracheally inoculated into corresponding mice at 48 h and 24 h before viral challenge. (**b)** One dose of DIG-4 (5 µg/mouse), PAD4 (5 µg/mouse), DIG-3 (5 µg/mouse), or PHW (5µg/mouse) packaged by TAT-P1 were intratracheally inoculated into corresponding mice at 24 h before viral challenge. Body weight (%) was the body weight of each day normalized to the body weight of Day 0. Data were presented as mean ± SD of five mice.

**a**

**b**


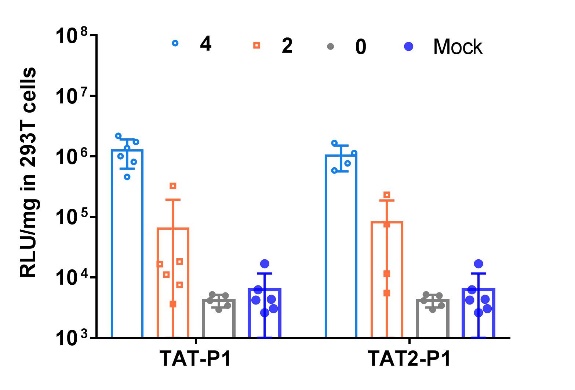

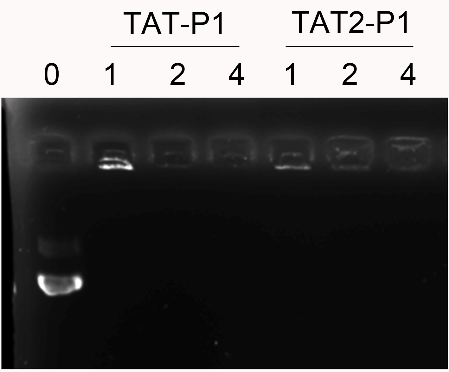


**Supplementary Fig. 4.** **The ability of TAT-P1 and TAT2-P1 binding to DNA determined by gel retardation assay and the transfection efficiency of peptide vectors in 293T cells.** (**a**) Peptides (TAT-P1, and TAT2-P1) were mixed with plasmid DNA in the indicated weight ratios (0, 1, 2, 4) of peptide:DNA at room temperature for 15 min. The peptide-DNA complexes were loaded into agarose gel for electrophoresis. (**b**) The transfection efficiency of TAT-P1 and TAT2-P1 in 293T cells (n = 4). The peptide:DNA complexes were mixed as the indicated ratio (4, 2, 0). The transfection efficiency was measured by luciferase assay. *P* values were calculated by the two-tailed Student’s *t* test when compared with mock. Data were presented as mean ± SD of at least four biological samples.

**Supplementary Fig. 5. Body weight changes of mice inoculated with vectors.** Mice were intratracheally inoculated with TAT-P1/DNA or TAT2-P1/DNA (20 μg/5 μg) or untreated mice (Mock). The body weight was normalized to the body weight of day 0 to generate the percentage of body weight. * indicates *P* < 0.05. *P* value was calculated by the two-tailed Student’s *t* test by comparing TAT2-P1 with TAT-P1. Data were presented as mean ± SD of at least three mice in each group.


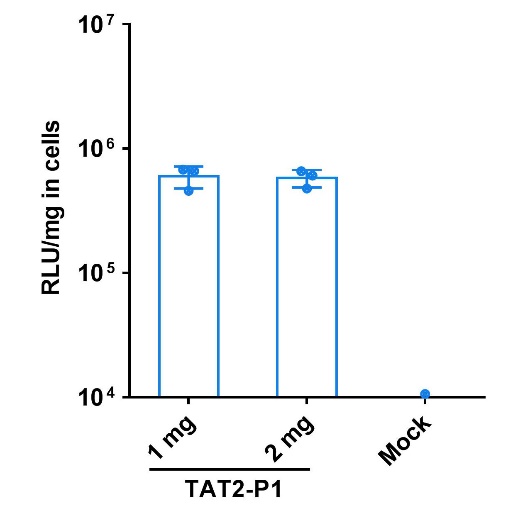


**Supplementary Fig. 6. The transfection efficiency of TAT2-P1 in 293T cells.** The plasmid DNA (pLuciferase) was packaged by TAT2-P1 (1 mg ml^-1^) or TAT2-P1 (2 mg ml^-1^) with the weight ratio (4:1) of peptide:DNA. The same amount of TAT2-P1/DNA was transfected to 293T cells. Luciferase expression was measured at 24 h post transfection. Mock means cells treated with TAT2-P1 without DNA. Data were presented as mean ± SD of three biological experiments.


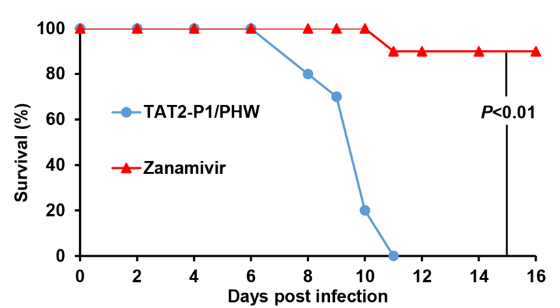


**Supplementary Fig. 7. The protection efficiency of zanamivir on A(H1N1)pdm09-infected mice for 1-day prophylactic.** Zanamivir (40 µg/mouse) was inoculated to mouse lungs at 1-day before A(H1N1)pdm09 virus challenge. The survivals were generated from 10 mice in each group. *P* value was calculated by Gehan–Breslow–Wilcoxon test.

**
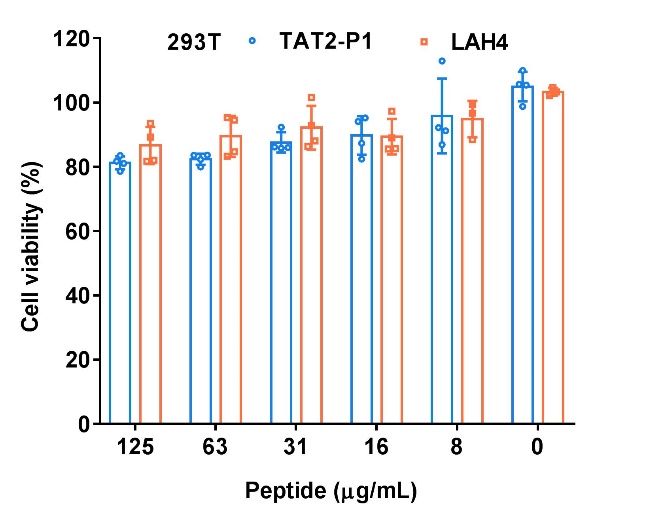
**

**Supplementary Fig. 8. Cytotoxicity of TAT2-P1 and LAH4 in 293T cells.** The indicated concentrations of peptides were incubated into 293T cells for 24 h. Cell viability (%) was the OD value of peptide-treated cells normalized to that of cells without treatment. The cytotoxicity was measured by MTT assay. Data were presented as mean ± SD of four biological samples.


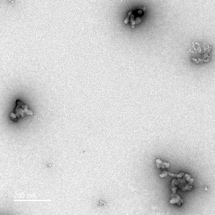


**3:2**

**TAT2-P1&LAH4**

**4:1**

**9:1**


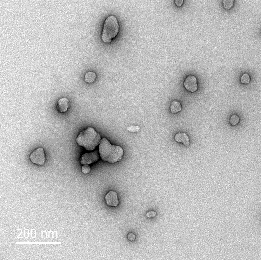

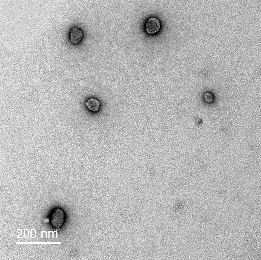


LAH4


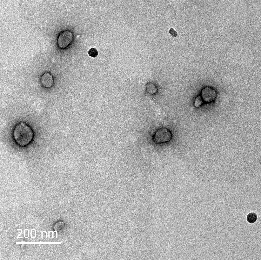


**Supplementary Fig. 9. TAT2-P1&LAH4 could form small spherical nanoparticles.** The pCMV was packaged by LAH4 (1 mg ml^-1^), TAT2-P1&LAH4 (3:2), TAT2-P1&LAH4 (4:1), and TAT2-P1&LAH4 (9:1). Nanoparticles were negatively stained for Transmission Electron Microscopy analysis. Scale bars, 200 nm. Representative images were taken from two independent experiments.


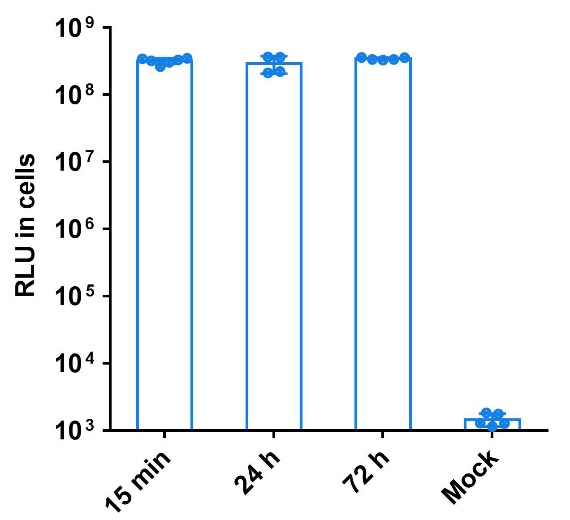


**Supplementary Fig. 10. The nanoparticle stability for cell transfection.** Luciferase plasmids packaged by TAT2-P1&LAH4 (4:1) were prepared for 72 h, 24 h and 15 min before the transfection in 293T cells. After transfection, the luciferase expression in cells were measured at 24 h post transfection. Data were presented as mean ± SD of at least four biological samples.


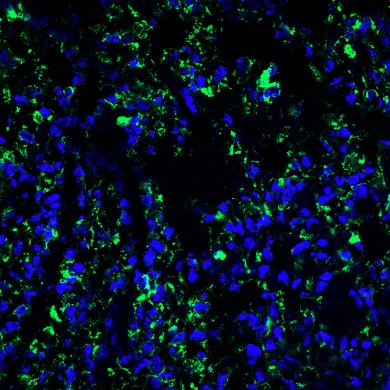

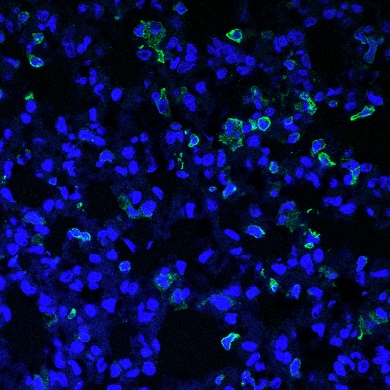


**Delta**

**PBS**

**CD3600**

**Mock**

NP

**a**

NP

**Omicron**

**Mock**

**CD3600**

**PBS**

**b**


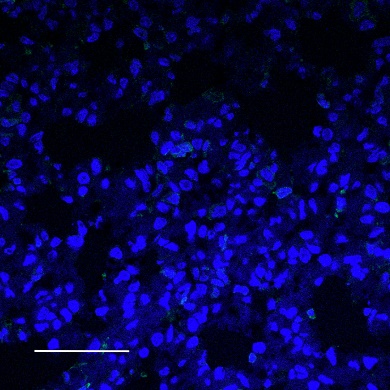

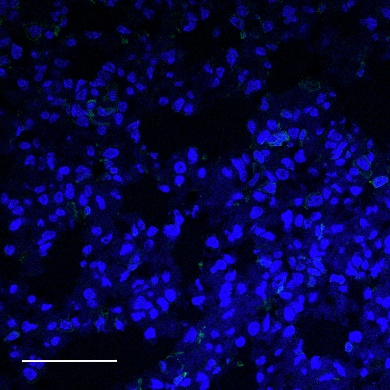

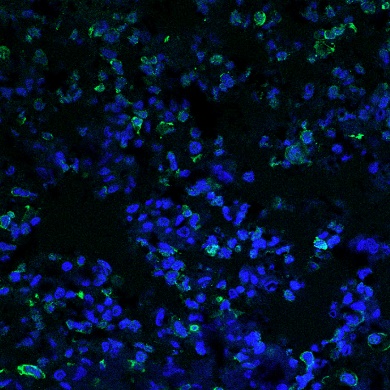

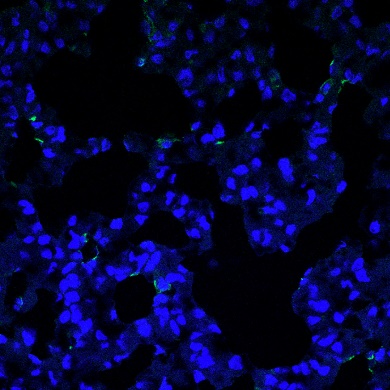


**Supplementary Fig. 11. The NP staining of hamster lungs.** (**a**) Hamsters were infected with Delta variant and treated with two doses of PBS or TAT2-P1&LAH4/CD3600. The infected lungs were collected at day 2 post infection for NP staining. (**b**) Hamsters were infected with Omicron variant and treated with two doses of PBS or TAT2-P1&LAH4/CD3600. The infected lungs were collected at day 2 post infection for NP staining (NP: green). Blue: nuclei. Mock, the non-infected lungs. Scale bar = 50 μm.

**Supplementary Table 1: Sequences of SARS-CoV-2 DIGs.**

| Gene | Oligonucleotide sequence (5' to3') |
| --- | --- |
| *CD2100* | ATTAAAGGTTTATACCTTCCCAGGTAACAAACCAACCAACTTTCGATCTCTTGTAGATCTGTTCTCTAAACGAACTTTAAAATCTGTGTGGCTGTCACTCGGCTGCATGCTTAGTGCACTCACGCAGTATAATTAATAACTAATTACTGTCGTTGACAGGACACGAGTAACTCGTCTATCTTCTGCAGGCTGCTTACGGTTTCGTCCGTGTTGCAGCCGATCATCAGCACATCTAGGTTTCGTCCGGGTGTGACCGAAAGGTAAGATGGAGAGCCTTGTCCCTGGTTTCAACGAGAAAACACACGTCCAACTCAGTTTGCCTGTTTTACAGGTTCGCGACGTGCTCGTACGTGGCTTTGGAGACTCCGTGGAGGAGGTCTTATCAGAGGCACGTCAACATCTTAAAGATGGCACTTGTGGCTTAGTAGAAGTTGAAAAAGGCGTTTTGCCTCAACTTGAACAGCCCTATGTGTTCATCAAACGTTCGGATGCTCGAACTGCACCTCATGGTCATGTTATGGTTGAGCTGGTAGCAGAACTCGAAGGCATTCAGTACGGTCGTAGTGGTGAGACACTTGGTGTCCTTGTCCCTCATGTGGGCGAAATACCAGTGGCTTACCGCAAGGTTCTTCTTCGTAAGAACGGTAATAAAGGAGCTGGTGGCCATAGTTACGGCGCCGATCTAAAGTCATAACAGGGTGAAGTACCAGTTTCTATCATTAATAACACTGTTTACACAAAAGTTGATGGTGTTGATGTAGAATTGTTTGAAAATAAAACAACATTACCTGTTAATGTAGCATTTGAGCTTTGGGCTAAGCGCAACATTAAACCAGTACCAGAGGTGAAAATACTCAATAATTTGGGTGTGGACATTGCTGCTAATACTGTGATCTGGGACTACAAAAGAGATGCTCCAGCACATATATCTACTATTGGTGTTTGTTCTATGACTGACATAGCCAAGAAACCAACTGAAACGATTTGTGCACCACTCACTGTCTTTTTTGATGGTAGAGTTGATGGTCAAGTAGACTTATTTAGAAATGCCCGTAATGGTGTTCTTATTACAGAAGGTAGTGTTAAAGGTTTACAACCATCTGTAGGTCCCAAACAAGCTAGTCTTAATGGAGTCACATTAATTGGAGAAGCCGTAAAAACACAGTTCAATTATTATAAGAAAGTTGATGGTGTTGTCCAACAATTACCTGAAACTTACTTTACTCAGAGTAGAAATTTACAAGAATTTAAACCCAGGAGTCAAATGGAAATTGATTTCTTAGAATTAGCTATGGATGAATTCATTGAACGGTATAAATTAGAAGGCTATGCCTTCGAACATATCGTTTATGGAGATTTTAGTCATAGTCAGTTAGGTGGTTTACATCTACTGATTGGACTAGCTAAACGTTTTAAGGAATCCGTTCTTCGGAATGTCGCGCATTGGCATGGAAGTCACACCTTCGGGAACGTGGTTGACCTACACAGGTGCCATCAAATTGGATGACAAAGATCCAAATTTCAAAGATCAAGTCATTTTGCTGAATAAGCATATTGACGCATACAAAACATTCCCACCAACAGAGCCTAAAAAGGACAAAAAGAAGAAGGCTGATGAAACTCAAGCCTTACCGCAGAGACAGAAGAAACAGCAAACTGTGACTCTTCTTCCTGCTGCAGATTTGGATGATTTCTCCAAACAATTGCAACAATCCATGAGCAGTGCTGACTCAACTCAGGCCTAAACTCATGCAGACCACACAAGGCAGATGGGCTATATAAACGTTTTCGCTTTTCCGTTTACGATATATAGTCTACTCTTGTGCAGAATGAATTCTCGTAACTACATAGCACAAGTAGATGTAGTTAACTTTAATCTCACATAGCAATCTTTAATCAGTGTGTAACATTAGGGAGGACTTGAAAGAGCCACCACATTTTCACCGAGGCCACGCGGAGTACGATCGAGTGTACAGTGAACAATGCTAGGGAGAGCTGCCTATATGGAAGAGCCCTAATGTGTAAAATTAATTTTAGTAGTGCTATCCCCATGTGATTTTAATAGCTTCTTAGGAGAATGACAAAAAAAAAAAAAAAAAAAA |
| *CD3600* | ATTAAAGGTTTATACCTTCCCAGGTAACAAACCAACCAACTTTCGATCTCTTGTAGATCTGTTCTCTAAACGAACTTTAAAATCTGTGTGGCTGTCACTCGGCTGCATGCTTAGTGCACTCACGCAGTATAATTAATAACTAATTACTGTCGTTGACAGGACACGAGTAACTCGTCTATCTTCTGCAGGCTGCTTACGGTTTCGTCCGTGTTGCAGCCGATCATCAGCACATCTAGGTTTCGTCCGGGTGTGACCGAAAGGTAAGATGGAGAGCCTTGTCCCTGGTTTCAACGAGAAAACACACGTCCAACTCAGTTTGCCTGTTTTACAGGTTCGCGACGTGCTCGTACGTGGCTTTGGAGACTCCGTGGAGGAGGTCTTATCAGAGGCACGTCAACATCTTAAAGATGGCACTTGTGGCTTAGTAGAAGTTGAAAAAGGCGTTTTGCCTCAACTTGAACAGCCCTATGTGTTCATCAAACGTTCGGATGCTCGAACTGCACCTCATGGTCATGTTATGGTTGAGCTGGTAGCAGAACTCGAAGGCATTCAGTACGGTCGTAGTGGTGAGACACTTGGTGTCCTTGTCCCTCATGTGGGCGAAATACCAGTGGCTTACCGCAAGGTTCTTCTTCGTAAGAACGGTAATAAAGGAGCTGGTGGCCATAGTTACGGCGCCGATCTAAAGTCATTTGACTTAGGCGACGAGCTTGGCACTGATCCTTATGAAGATTTTCAAGAAAACTGGAACACTAAACATAGCAGTGGTGTTACCCGTGAACTCATGCGTGAGCTTAACGGAGGGGCATACACTCGCTATGTCGATAACAACTTCTGTGGCCCTGATGGCTACCCTCTTGAGTGCATTAAAGACCTTCTAGCACGTGCTGGTAAAGCTTCATGCACTTTGTCCGAACAACTGGACTTTATTGACACTAAGAGGGGTGTATACTGCTGCCGTGAACATGAGCATGAAATTGCTTGGTACACGGAACGTTCTGAAAAGAGCTATGAATTGCAGACACCTTTTGAAATTAAATTGGCAAAGAAATTTGACACCTTCAATGGGGAATGTCCAAATTTTGTATTTCCCTTAAATTCCATAATCAAGACTATTCAACCAAGGGTTGAAAAGAAAAAGCTTGATGGCTTTATGGGTAGAATTCGATCTGTCTATCCAGTTGCGTCACCAAATGAATGAAAGTCTGCTACGTGTATAACACGTTGCAATTTAGGTGGTGCTGTCTGTAGACATCATGCTAATGAGTACAGATTGTATCTCGATGCTTATAACATGATGATCTCAGCTGGCTTTAGCTTGTGGGTTTACAAACAATTTGATACTTATAACCTCTGGAACACTTTTACAAGACTTCAGAGTTTAGAAAATGTGGCTTTTAATGTTGTAAATAAGGGACACTTTGATGGACAACAGGGTGAAGTACCAGTTTCTATCATTAATAACACTGTTTACACAAAAGTTGATGGTGTTGATGTAGAATTGTTTGAAAATAAAACAACATTACCTGTTAATGTAGCATTTGAGCTTTGGGCTAAGCGCAACATTAAACCAGTACCAGAGGTGAAAATACTCAATAATTTGGGTGTGGACATTGCTGCTAATACTGTGATCTGGGACTACAAAAGAGATGCTCCAGCACATATATCTACTATTGGTGTTTGTTCTATGACTGACATAGCCAAGAAACCAACTGAAACGATTTGTGCACCACTCACTGTCTTTTTTGATGGTAGAGTTGATGGTCAAGTAGACTTATTTAGAAATGCCCGTAATGGTGTTCTTATTACAGAAGGTAGTGTTAAAGGTTTACAACCATCTGTAGGTCCCAAACAAGCTAGTCTTAATGGAGTCACATTAATTGGAGAAGCCGTAAAAACACAGTTCAATTATTATAAGAAAGTTGATGGTGTTGTCCAACAATTACCTGAAACTTACTTTACTCAGAGTAGAAATTTACAAGAATTTAAACCCAGGAGTCAAATGGAAATTGATTTCTTAGAATTAGCTATGGATGAATTCATTGAACGGTATAAATTAGAAGGCTATGCCTTCGAACATATCGTTTATGGAGATTTTAGTCATAGTCAGTTAGGTGGTTTACATCTACTGATTGGACTAGCTAAACGTTTTAAGGAATCACCTTTTGAATTAGAAGATTTTATTCCTATGGACAGTACAGTTAAAAACTATTTCATAACAGATGCGCAAACAGGTTCATCTAAGTGTGTGTGTTCTGTTATTGATTTATTACTTGATGATTTTGTTGAAATAATAAAATCCCAAGATTTATCTGTAGTTTCTAAGGTTGTCAAAGTGACTATTGACTATACAGAAATTTCATTTATGCTTTGGTGTAAAGATGGCCATGTAGAAACATTATACACCAAAAGATCACATTGGCACCCGCAATCCTGCTAACAATGCTGCAATCGTGCTACAACTTCCTCAAGGAACAACATTGCCAAAAGGCTTCTACGCAGAAGGGAGCAGAGGCGGCAGTCAAGCCTCTTCTCGTTCCTCATCACGTAGTCGCAACAGTTCAAGAAATTCAACTCCAGGCAGCAGTAGGGGAACTTCTCCTGCTAGAATGGCTGGCAATGGCGGTGATGCTGCTCTTGCTTTGCTGCTGCTTGACAGATTGAACCAGCTTGAGAGCAAAATGTCTGGTAAAGGCCAACAACAACAAGGCCAAACTGTCACTAAGAAATCTGCTGCTGAGGCTTCTAAGAAGCCTCGGCAAAAACGTACTGCCACTAAAGCATACAATGTAACACAAGCTTTCGGCAGACGTGGTCCAGAACAAACCCAAGGAAATTTTGGGGACCAGGAACTAATCAGACAAGGAACTGATTACAAACATTGGCCGCAAATTGCACAATTTGCCCCCAGCGCTTCAGCGTTCTTCGGAATGTCGCGCATTGGCATGGAAGTCACACCTTCGGGAACGTGGTTGACCTACACAGGTGCCATCAAATTGGATGACAAAGATCCAAATTTCAAAGATCAAGTCATTTTGCTGAATAAGCATATTGACGCATACAAAACATTCCCACCAACAGAGCCTAAAAAGGACAAAAAGAAGAAGGCTGATGAAACTCAAGCCTTACCGCAGAGACAGAAGAAACAGCAAACTGTGACTCTTCTTCCTGCTGCAGATTTGGATGATTTCTCCAAACAATTGCAACAATCCATGAGCAGTGCTGACTCAACTCAGGCCTAAACTCATGCAGACCACACAAGGCAGATGGGCTATATAAACGTTTTCGCTTTTCCGTTTACGATATATAGTCTACTCTTGTGCAGAATGAATTCTCGTAACTACATAGCACAAGTAGATGTAGTTAACTTTAATCTCACATAGCAATCTTTAATCAGTGTGTAACATTAGGGAGGACTTGAAAGAGCCACCACATTTTCACCGAGGCCACGCGGAGTACGATCGAGTGTACAGTGAACAATGCTAGGGAGAGCTGCCTATATGGAAGAGCCCTAATGTGTAAAATTAATTTTAGTAGTGCTATCCCCATGTGATTTTAATAGCTTCTTAGGAGAATGACAAAAAAAAAAAAAAAAAAAAA |

**Supplementary Table 2: Sequences of influenza DIGs.**

| Gene | Oligonucleotide sequence (5' to3') |
| --- | --- |
| *PAD1* | AGCGAAAGCAGGTCAATTATATTCAATATGGAAAGAATAAAAGAACTAAGGAATCTAATGTCGCAGTCTCGCACTCGCGAGATACTCACAAAAACCACCGTGGACCATATGGCCATAATCAAGAAGTACACATCAGGAAGACAGGAGAAGAAGGAGAGAAGGCTAATGTGCTAATTGGGAAGGAGACGTGGTGTTGGTAATGAAACGGAAACGGAACTCTAGCATACTTACTGACAGCCAGACAGCGACCAAAAGAATTCGGATGGCCATCAATTAGTGTCGAATAGTTTAAAAACGACCTTGTTTCTACT |
| *PB1D1* | AGCGAAAGCAGGCAAACCATTTGAATGGATGTCAATCCGACTTTACTTTTCTTAAAAGTGCCAGCACAAAATGCTATAAGCACAACTTTCCCTTATACTGGAGACCCTCCTTACAGCCATGGGACAGGAACAGGATACACCATGGATACTGTCATTCCAGAGCCCGAATTGATGCACGAATTGATTTCGAATCTGGAAGGATAAAGAAAGAGGAGTTCACTGAGATCATGAAGATCTGTTCCACCATTGAAGAGCTCAGACGGCAAAAATAGTGAATTTAGCTTGTCCTTCATGAAAAAATGCCTTGTTTCTACT |
| *PB2D1* | AGCGAAAGCAGGTACTGATTCAAAATGGAAGATTTTGTGCGACAATGCTTCAATCCGATGATTGTCGAGCTTGCGGAAAAGGCAATGAAAGAGTATGGAGAGGACCTGAAAATCGAAACAAACAAATTTGCAGCAATATGCACTCACTTGGAAGTGGCTATATGAAGCAATTGAGGAGTGCCTGATTAATGATCCCTGGGTTTTGCTTAATGCTTCTTGGTTCAACTCCTTCCTCACACATGCATTGAGATAGTTGTGGCAATGCTACTATTTGCTATCCATACTGTCCAAAAAAGTACCTTGTTTCTACT |
| *PAD2* | AGCGAAAGCAGGTACTGATTCAAAATGGAAGATTTTGTGCGACAATGCTTCAATCCGATGATTGTCGAGCTTGCGGAAAAGGCAATGAAAGAGTATGGAGAGGACCTGAAAATCGAAACAAACAAATTTGCAGCAATATGCACTCACTTGGAAGTGTGCTTCATGTATTCAGATTTTCACTTCATCGATGAGCAAGGCGAGTCAATAGTCGTAGAACTTGGCGATCAAGAAAACTGCTTCTTATCGTTCAGGCTCTTAGGGACAACCTGGAACCTGGGACCTTTGATCTTGGGGGGCTATATGAAGCAATTGAGGAGTGCCTGATTAATGATCCCTGGGTTTTGCTTAATGCTTCTTGGTTCAACTCCTTCCTCACACATGCATTGAGATAGTTGTGGCAATGCTACTATTTGCTATCCATACTGTCCAAAAAAGTACCTTGTTTCTACT |
| *PB1D2* | AGCGAAAGCAGGCAAACCATTTGAATGGATGTCAATCCGACTTTACTTTTCTTAAAAGTGCCAGCACAAAATGCTATAAGCACAACTTTCCCTTATACTGGAGACCCTCCTTACAGCCATGGGACAGGAACAGGATACACCATGGATACTGTCAACAGGACACATCAGTACTCAGAAAGGGGAAGATGGACAACAAACACCGAAACTGGAGCACCGCAACTCAACAATTCTTCCCCAGCAGTTCATACAGAAGACCAGTCGGGATATCCAGTATGGTGGAGGCTATGGTTTCCAGAGCCCGAATTGATGCACGAATTGATTTCGAATCTGGAAGGATAAAGAAAGAGGAGTTCACTGAGATCATGAAGATCTGTTCCACCATTGAAGAGCTCAGACGGCAAAAATAGTGAATTTAGCTTGTCCTTCATGAAAAAATGCCTTGTTTCTACT |
| *PB2D2* | AGCGAAAGCAGGTCAATTATATTCAATATGGAAAGAATAAAAGAACTAAGGAATCTAATGTCGCAGTCTCGCACTCGCGAGATACTCACAAAAACCACCGTGGACCATATGGCCATAATCAAGAAGTACACATCAGGAAGACAGGAGAAGAACCCAGCACTTAGGATGAAATGGATGATGGCAATGAAATATCCAATTACAGCAGACAAGAGGATAACGGAAATGGGGCAAAGAAGACAGGAGATATGGACCAGCATTAAGCATAAATGAACTGAGCAACCTTGCGAAAGGAGAGAAGGCTAATGTGCTAATTGGGCAAGGAGACGTGGTGTTGGTAATGAAACGGAAACGGAACTCTAGCATACTTACTGACAGCCAGACAGCGACCAAAAGAATTCGGATGGCCATCAATTAGTGTCGAATAGTTTAAAAACGACCTTGTTTCTACT |
| *PAD3* | AGCAAAAGCAGGTACTGATTCAAAATGGAAGATTTTGTGCGACAATGCTTCAATCCGATGATTGTCGAGCTTGCGGAAAAGGCAATGAAAGAGTATGGAGAGGACCTGAAAATCGAAACAAACAAATTTGCAGCAATATGCACTCACTTGGAAGTGTGCTTCATGTATTCAGATTTTCACTTCATCGATGAGCAAGGCGAGTCAATAGTCGTAGAACTTGGCGATCCAAATGCACTTTTGAAGCACAGATTTGAAATAATCGAGGGAAGAGATCGCACAATAGCCTGGACAGTAATAAACAGTATTTGCAACACTACAGGGGCTGAGAAACCAAAGTTTCTACCAGATTTGTATGAGAGAGTCCCCCAAAGGAGTGGAGGAAGGTTCCATTGGGAAGGTCTGCAGAACTTTATTGGCAAAGTCGGTATTCAACAGCTTGTATGCATCTCCACAACTGGAAGGATTTTCAGCTGAATCAAGAAAACTGCTTCTTATCGTTCAGGCTCTTAGGGACAACCTGGAACCTGGGACCTTTGATCTTGGGGGGCTATATGAAGCAATTGAGGAGTGCCTGATTAATGATCCCTGGGTTTTGCTTAATGCTTCTTGGTTCAACTCCTTCCTCACACATGCATTGAGATAGTTGTGGCAATGCTACTATTTGCTATCCATACTGTCCAAAAAAGTACCTTGTTTCTACT |
| *PB1D3* | AGCAAAAGCAGGCAAACCATTTGAATGGATGTCAATCCGACTTTACTTTTCTTAAAAGTGCCAGCACAAAATGCTATAAGCACAACTTTCCCTTATACTGGAGACCCTCCTTACAGCCATGGGACAGGAACAGGATACACCATGGATACTGTCAACAGGACACATCAGTACTCAGAAAGGGGAAGATGGACAACAAACACCGAAACTGGAGCACCGCAACTCAACCCGATTGATGGGCCACTGCCAGAAGACAATGAACCAAGTGGTTATGCCCAAACAGATCAAAAGAAATCGATCCATCTTGAATACAAGCCAAAGAGGAATACTTGAAGATGAACAAATGTACCAAAAGTGCTGCAACTTATTTGAAAAATTCTTCCCCAGCAGTTCATACAGAAGACCAGTCGGGATATCCAGTATGGTGGAGGCTATGGTTTCCAGAGCCCGAATTGATGCACGAATTGATTTCGAATCTGGAAGGATAAAGAAAGAGGAGTTCACTGAGATCATGAAGATCTGTTCCACCATTGAAGAGCTCAGACGGCAAAAATAGTGAATTTAGCTTGTCCTTCATGAAAAAATGCCTTGTTTCTACT |
| *PB2D3* | AGCGAAAGCAGGTCAATTATATTCAATATGGAAAGAATAAAAGAACTAAGGAATCTAATGTCGCAGTCTCGCACTCGCGAGATACTCACAAAAACCACCGTGGACCATATGGCCATAATCAAGAAGTACACATCAGGAAGACAGGAGAAGAACCCAGCACTTAGGATGAAATGGATGATGGCAATGAAATATCCAATTACAGCAGACAAGAGGATAACGGAAATGATTCCTGAGAGAAATGAGCAGGGACAAACTTTATGGAGTAAAATGAATGACGCCGGATCAGACCGAGTGATGGTATCACCTAACTGAAGACCCAGATGAAGGCACAGCTGGAGTTGAGTCCGCAGTTCTGAGAGGATTCCTCATTCTGGGCAAAGAAGACAGGAGATATGGACCAGCATTAAGCATAAATGAACTGAGCAACCTTGCGAAAGGAGAGAAGGCTAATGTGCTAATTGGGCAAGGAGACGTGGTGTTGGTAATGAAACGGAAACGGAACTCTAGCATACTTACTGACAGCCAGACAGCGACCAAAAGAATTCGGATGGCCATCAATTAGTGTCGAATAGTTTAAAAACGACCTTGTTTCTACT |
|  |  |

**Supplementary Table 3: Sequences of influenza DIGs.**

| Gene | Oligonucleotide sequence (5' to3') |
| --- | --- |
| *PAD4* | AGCGAAAGCAGGTACTGATTCAAAATGGAAGATTTTGTGCGACAATGCTTCAATCCGATGATTGTCGAGCTTGCGGAAAAGGCAATGAAAGAGTATGGAGAGGACCTGAAAATCGAAACAAACAAATTTGCAGCAATATGCACTCACTTGGAAGTGTGCTTCATGTATTCAGATTTTCACTTCATCGATGAGCAAGGCGAGTCAATAGTCGTAGAACTTGGCGATCCAAATGCACTTTTGAAGCACAGATTTGAAATAATCGAGGGAAGAGATCGCACAATAGCCTGGACAGTAATAAACAGTATTTGCAACACTACAGGGGCTGAGAAACCAAAGTTTCTACCAGATTTGTATGATTACAAGAAGAATAGATTCATCGAAATTGGAGTAACAAGGAGAGAAGTTCACATATACTATCTGGAAAAGGCCAATAAAATTAAATCTGAGAAGACACACATCCACACCTCCTTCAGTCACTTCAACAAATCGAGAGTATGATTGAAGCTGAGTCCTCTGTCAAGGAGAAAGACATGACCAAAGAGTTCTTTGAAAACAAATCAGAAACATGGCCCGTTGGAGAGTCCCCCAAAGGAGTGGAGGAAGGTTCCATTGGGAAGGTCTGCAGAACTTTATTGGCAAAGTCGGTATTCAACAGCTTGTATGCATCTCCACAACTGGAAGGATTTTCAGCTGAATCAAGAAAACTGCTTCTTATCGTTCAGGCTCTTAGGGACAACCTGGAACCTGGGACCTTTGATCTTGGGGGGCTATATGAAGCAATTGAGGAGTGCCTGATTAATGATCCCTGGGTTTTGCTTAATGCTTCTTGGTTCAACTCCTTCCTCACACATGCATTGAGATAGTTGTGGCAATGCTACTATTTGCTATCCATACTGTCCAAAAAAGTACCTTGTTTCTACT |
| *PB1D4* | AGCGAAAGCAGGCAAACCATTTGAATGGATGTCAATCCGACTTTACTTTTCTTAAAAGTGCCAGCACAAAATGCTATAAGCACAACTTTCCCTTATACTGGAGACCCTCCTTACAGCCATGGGACAGGAACAGGATACACCATGGATACTGTCAACAGGACACATCAGTACTCAGAAAGGGGAAGATGGACAACAAACACCGAAACTGGAGCACCGCAACTCAACCCGATTGATGGGCCACTGCCAGAAGACAATGAACCAAGTGGTTATGCCCAAACAGATTGTGTATTGGAAGCAATGGCCTTCCTTGAGGAATCCCATCCTGGTATCTTTGAGACCTCGTGTCTTGAAACGATGGAGGTTGTTCAGCAAACACGAGTGGACAAGCTGACACAAGGCCGACAGACCTATGACTGGACTCTAAATAGGAACCAGCCTGCTGCAACAGCGGATTACCAGGGGCGTTTATGCAACCCACTGAACCCATTTGTCAACCATAAAGACATTGAATCAGTGAACAATGCAGTGATAATGCCAGCACATGGTCCAGCCAAAAACATGGAGTATGATGCTGTTGCAACAACACACTCCTGGATCCCCAAAAGAAATCGATCCATCTTGAATACAAGCCAAAGAGGAATACTTGAAGATGAACAAATGTACCAAAAGTGCTGCAACTTATTTGAAAAATTCTTCCCCAGCAGTTCATACAGAAGACCAGTCGGGATATCCAGTATGGTGGAGGCTATGGTTTCCAGAGCCCGAATTGATGCACGAATTGATTTCGAATCTGGAAGGATAAAGAAAGAGGAGTTCACTGAGATCATGAAGATCTGTTCCACCATTGAAGAGCTCAGACGGCAAAAATAGTGAATTTAGCTTGTCCTTCATGAAAAAATGCCTTGTTTCTACT |
| *PB2D4* | AGCGAAAGCAGGTCAATTATATTCAATATGGAAAGAATAAAAGAACTAAGGAATCTAATGTCGCAGTCTCGCACTCGCGAGATACTCACAAAAACCACCGTGGACCATATGGCCATAATCAAGAAGTACACATCAGGAAGACAGGAGAAGAACCCAGCACTTAGGATGAAATGGATGATGGCAATGAAATATCCAATTACAGCAGACAAGAGGATAACGGAAATGATTCCTGAGAGAAATGAGCAGGGACAAACTTTATGGAGTAAAATGAATGACGCCGGATCAGACCGAGTGATGGTATCACCTCTGGCTGTGACATGGTGGAATAGGAATGGACCAGTGACAAGTACAGTTCATTATCCAAAAATCTACAAAACTTATTTTGAAAAAGTCGAAAGGTTAAAACATGGAACCTTTGGCCCTGTCCATTTTAGAAACCAAGTCAAAATACACTTCTTCCCTTCGCAGCCGCTCCACCAAAGCAAAGTGGAATGCAGTTCTCCTCATTGACTATAAATGTGAGGGGATCAGGAATGAGAATACTTGTAAGGGGCAATTCTCCAATATTCAACTACAACAAGACCACTAAAAGACTCACAGTTCTCGGAAAGGATGCTGGCCCTTTAACTGAAGACCCAGATGAAGGCACAGCTGGAGTTGAGTCCGCAGTTCTGAGAGGATTCCTCATTCTGGGCAAAGAAGACAGGAGATATGGACCAGCATTAAGCATAAATGAACTGAGCAACCTTGCGAAAGGAGAGAAGGCTAATGTGCTAATTGGGCAAGGAGACGTGGTGTTGGTAATGAAACGGAAACGGAACTCTAGCATACTTACTGACAGCCAGACAGCGACCAAAAGAATTCGGATGGCCATCAATTAGTGTCGAATAGTTTAAAAACGACCTTGTTTCTACT |
| *PAD5* | AGCGAAAGCAGGTACTGATTCAAAATGGAAGATTTTGTGCGACAATGCTTCAATCCGATGATTGTCGAGCTTGCGGAAAAGGCAATGAAAGAGTATGGAGAGGACCTGAAAATCGAAACAAACAAATTTGCAGCAATATGCACTCACTTGGAAGTGTGCTTCATGTATTCAGATTTTCACTTCATCGATGAGCAAGGCGAGTCAATAGTCGTAGAACTTGGCGATCCAAATGCACTTTTGAAGCACAGATTTGAAATAATCGAGGGAAGAGATCGCACAATAGCCTGGACAGTAATAAACAGTATTTGCAACACTACAGGGGCTGAGAAACCAAAGTTTCTACCAGATTTGTATGATTACAAGAAGAATAGATTCATCGAAATTGGAGTAACAAGGAGAGAAGTTCACATATACTATCTGGAAAAGGCCAATAAAATTAAATCTGAGAAGACACACATCCACATTTTCTCATTCACTGGGGAGGAAATGGCCACAAAGGCCGACTACACTCTCGATGAAGAAAGCAGGGCTAGGATCAAAACCAGGCTATTCACCATAAGACAAGAAATGGCTAGCAGAGGCCTCTGGGATTCCTTTCGTCAGTCAATGGGAGAAGTACTGTGTTCTTGAGGTAGGAGATATGCTTCTAAGAAGTGCCATAGGCCATGTGTCAAGGCCTATGTTCTTGTATGTGAGGACAAATGGAACCTCAAAAATTAAAATGAAATGGGGGATGGAAATGAGGCGTTGCCTCCTTCAGTCACTTCAACAAATCGAGAGTATGATTGAAGCTGAGTCCTCTGTCAAGGAGAAAGACATGACCAAAGAGTTCTTTGAAAACAAATCAGAAACATGGCCCGTTGGAGAGTCCCCCAAAGGAGTGGAGGAAGGTTCCATTGGGAAGGTCTGCAGAACTTTATTGGCAAAGTCGGTATTCAACAGCTTGTATGCATCTCCACAACTGGAAGGATTTTCAGCTGAATCAAGAAAACTGCTTCTTATCGTTCAGGCTCTTAGGGACAACCTGGAACCTGGGACCTTTGATCTTGGGGGGCTATATGAAGCAATTGAGGAGTGCCTGATTAATGATCCCTGGGTTTTGCTTAATGCTTCTTGGTTCAACTCCTTCCTCACACATGCATTGAGATAGTTGTGGCAATGCTACTATTTGCTATCCATACTGTCCAAAAAAGTACCTTGTTTCTACT |
|  |  |

**Supplementary Table 4: Peptide sequences.**

| Peptide | Peptide sequence |
| --- | --- |
| TAT-P1 | YGRKKRRQRRRCWGPCPTAFRQIGNCGRFRVRCCRIR |
| TAT2-P1 | RKKRRQRRRCWGPCPTAFRQIGNCGRFRVRCCRIR |
| P1 | CWGPCPTAFRQIGNCGRFRVRCCRIR |
| P9R | NGAICWGPCPTAFRQIGNCGRFRVRCCRIR |
| LAH4 | KKALLAHALHLLALLALHLAHALKKA-NH2 |

**Supplementary Table 5: Primers for RT-qPCR.**

| Gene | Primer | Oligonucleotide sequence (5' to3') |
| --- | --- | --- |
| *PAD4* | DI-PA-F | ATCTGAGAAGACACACATCCAC |
|  | DI-PA-R | GGACTCTCCAACGGGCCATGTT |
| *PB1D3* | DI-PB1-F | CACCGAAACTGGAGCACCGCAAC |
|  | DI-PB1-R | TTTGTTCATCTTCAAGTATTCCT |
| *PB2D3* | DI-PB2-F | GATAACGGAAATGATTCCT |
|  | DI-PB2-R | TCAGAACTGCGGACTCAAC |
| *CD2100* | CD2100-F | CTTCGTAAGAACGGTAATAAAGG |
|  | CD2100-R | CAAACAATTCTACATCAACACC |
| *CD3600* | CD3600-F | ATAATCAAGACTATTCAACCAAGGG |
|  | CD3600-R | TCATTAGCATGATGTCTACAGAC |
| *SARS-CoV-2* | S-F | CCTACTAAATTAAATGATCTCTGCTTTACT |
|  | S-R | CAAGCTATAACGCAGCCTGTA |

**Supplementary Table 6: Primers for DIG construction.**

| Plasmid | Primer | Oligonucleotide sequence (5' to3') ^a^ | Restriction enzyme |
| --- | --- | --- | --- |
| PB2D1 | PB2-F | TATTGGTCTCAGGGAGCGAAAGCAGGTC | *Bsa*I |
|  | PB2-MR | GGTTCTTCTCCTGTCTTCCTG |  |
|  | PB2-MF | GAAGTACACATCAGGAAGACAGGAGAAGAACCGGAGAGAAGGCTAATGTGCTA |  |
|  | PB2-R | ATATGGTCTCGTATTAGTAGAAACAAGGTCGTTT | *Bsa*I |
| PB1D1 | PB1-F | TATTCGTCTCAGGGAGCAAAAGCAGGCA | *Bsm*BI |
|  | PB1-MR | TGACAGTATCCATGGTGTATC |  |
|  | PB1-MF | GGGACAGGAACAGGATACACCATGGATACTGTCATTCCAGAGCCCGAATTGATGC |  |
|  | PB1-R | ATATCGTCTCGTATTAGTAGAAACAAGGCATTT | *Bsm*BI |
| PAD1 | PA-F | TATTCGTCTCAGGGAGCAAAAGCAGGTAC | *Bsm*BI |
|  | PA-MR | ACTTCCAAGTGAGTGCATATTG |  |
|  | PA-MF | ACAAATTTGCAGCAATATGCACTCACTTGGAAGTGGCTATATGAAGCAATTGAG |  |
|  | PA-R | ATATCGTCTCGTATTAGTAGAAACAAGGTACTT | *Bsm*BI |
| PB2D2 | PB2-F | TATTGGTCTCAGGGAGCGAAAGCAGGTC | *Bsa*I |
|  | PB2-MR | CATTTCCGTTATCCTCTTGTCTG |  |
|  | PB2-MF | CCAATTACAGCAGACAAGAGGATAACGGAAATGGGGCAAAGAAGACAGGAGATATG |  |
|  | PB2-R | ATATGGTCTCGTATTAGTAGAAACAAGGTCGTTT | *Bsa*I |
| PB1D2 | PB1-F | TATTCGTCTCAGGGAGCAAAAGCAGGCA | *Bsm*BI |
|  | PB1-MR | GTTGAGTTGCGGTGCTCCAGTTTCG |  |
|  | PB1-MF | CAACAAACACCGAAACTGGAGCACCGCAACTCAACAATTCTTCCCCAGCAGTTCATAC |  |
|  | PB1-R | ATATCGTCTCGTATTAGTAGAAACAAGGCATTT | *Bsm*BI |
| PAD2 | PA-F | TATTCGTCTCAGGGAGCAAAAGCAGGTAC | *Bsm*BI |
|  | PA-MR | ATCGCCAAGTTCTACGACTATTG |  |
|  | PA-MF | GCAAGGCGAGTCAATAGTCGTAGAACTTGGCGATCAAGAAAACTGCTTCTTATCGT |  |
|  | PA-R | ATATCGTCTCGTATTAGTAGAAACAAGGTACTT | *Bsm*BI |
| PB2D3 | PB2-F | TATTGGTCTCAGGGAGCGAAAGCAGGTC | *Bsa*I |
|  | PB2-MR | GGTGATACCATCACTCGGTCTG |  |
|  | PB2-MF | CGCCGGATCAGACCGAGTGATGGTATCACCTAACTGAAGACCCAGATGAAGGC |  |
|  | PB2-R | ATATGGTCTCGTATTAGTAGAAACAAGGTCGTTT | *Bsa*I |
| PB1D3 | PB1-F | TATTCGTCTCAGGGAGCAAAAGCAGGCA | *Bsm*BI |
|  | PB1-MR | ATCTGTTTGGGCATAACCAC |  |
|  | PB1-MF | ACAATGAACCAAGTGGTTATGCCCAAACAGATCAAAAGAAATCGATCCATCTTGA |  |
|  | PB1-R | ATATCGTCTCGTATTAGTAGAAACAAGGCATTT | *Bsm*BI |
| PAD3 | PA-F | TATTCGTCTCAGGGAGCAAAAGCAGGTAC | *Bsm*BI |
|  | PA-MR | TCATACAAATCTGGTAGAAACTTTG |  |
|  | PA-MF | AGAAACCAAAGTTTCTACCAGATTTGTATGAGAGAGTCCCCCAAAGGAGTGGA |  |
|  | PA-R | ATATCGTCTCGTATTAGTAGAAACAAGGTACTT | *Bsm*BI |

**Supplementary Table 7: Primers for DIG construction.**

| Plasmid | Primer | Oligonucleotide sequence (5' to3') ^a^ | Restriction enzyme |
| --- | --- | --- | --- |
| PB2D4 | PB2-F | TATTGGTCTCAGGGAGCGAAAGCAGGTC | *Bsa*I |
|  | PB2-MR | GTATTTTGACTTGGTTTCTAA |  |
|  | PB2-MF | GCCCTGTCCATTTTAGAAACCAAGTCAAAATACACTTCTTCCCTTCGCAGCCGC |  |
|  | PB2-R | ATATGGTCTCGTATTAGTAGAAACAAGGTCGTTT | *Bsa*I |
| PB1D4 | PB1-F | TATTCGTCTCAGGGAGCAAAAGCAGGCA | *Bsm*BI |
|  | PB1-MR | GCTGTTGCAGCAGGCTGGTTC |  |
|  | PB1-MF | GGACTCTAAATAGGAACCAGCCTGCTGCAACAGCGGATTACCAGGGGCGTTTATG |  |
|  | PB1-R | ATATCGTCTCGTATTAGTAGAAACAAGGCATTT | *Bsm*BI |
| PAD4 | PA-F | TATTCGTCTCAGGGAGCAAAAGCAGGTAC | *Bsm*BI |
|  | PA-MR | TGTGGATGTGTGTCTTCTCAG |  |
|  | PA-MF | CAATAAAATTAAATCTGAGAAGACACACATCCACACCTCCTTCAGTCACTTCAAC |  |
|  | PA-R | ATATCGTCTCGTATTAGTAGAAACAAGGTACTT | *Bsm*BI |
| PAD5 | PA-F | TATTCGTCTCAGGGAGCAAAAGCAGGTAC | *Bsm*BI |
|  | PA-MR | GACTGACGAAAGGAATCCCAGAG |  |
|  | PA-MF | CTAGCAGAGGCCTCTGGGATTCCTTTCGTCAGTCAATGGGAGAAGTACTGTGTTC |  |
|  | PA-R | ATATCGTCTCGTATTAGTAGAAACAAGGTACTT | *Bsm*BI |
